# Chromatin Insulator Factors Involved in Long-Range DNA Interactions and Their Role in the Folding of the Drosophila Genome

**DOI:** 10.1371/journal.pgen.1004544

**Published:** 2014-08-28

**Authors:** Jutta Vogelmann, Antoine Le Gall, Stephanie Dejardin, Frederic Allemand, Adrien Gamot, Gilles Labesse, Olivier Cuvier, Nicolas Nègre, Martin Cohen-Gonsaud, Emmanuel Margeat, Marcelo Nöllmann

**Affiliations:** 1 Centre National de la Recherche Scientifique, Unité Mixte de Recherche 5048, Centre de Biochimie Structurale, Montpellier, France; 2 Institut National de la Santé et la Recherche Médicale, Unité 1054, Montpellier, France; 3 Universités Montpellier I et II, Montpellier, France; 4 Laboratoire de Biologie Moléculaire Eucaryote, CNRS and Université de Toulouse, Toulouse; France; 5 Laboratoire Diversité, Génomes & Interactions Microorganismes-Insectes, INRA UMR1333, Université de Montpellier 2, Montpellier, France; Max Planck Institute of Immunobiology and Epigenetics, Germany

## Abstract

Chromatin insulators are genetic elements implicated in the organization of chromatin and the regulation of transcription. In Drosophila, different insulator types were characterized by their locus-specific composition of insulator proteins and co-factors. Insulators mediate specific long-range DNA contacts required for the three dimensional organization of the interphase nucleus and for transcription regulation, but the mechanisms underlying the formation of these contacts is currently unknown. Here, we investigate the molecular associations between different components of insulator complexes (BEAF32, CP190 and Chromator) by biochemical and biophysical means, and develop a novel single-molecule assay to determine what factors are necessary and essential for the formation of long-range DNA interactions. We show that BEAF32 is able to bind DNA specifically and with high affinity, but not to bridge long-range interactions (LRI). In contrast, we show that CP190 and Chromator are able to mediate LRI between specifically-bound BEAF32 nucleoprotein complexes *in vitro*. This ability of CP190 and Chromator to establish LRI requires specific contacts between BEAF32 and their C-terminal domains, and dimerization through their N-terminal domains. In particular, the BTB/POZ domains of CP190 form a strict homodimer, and its C-terminal domain interacts with several insulator binding proteins. We propose a general model for insulator function in which BEAF32/dCTCF/Su(HW) provide DNA specificity (first layer proteins) whereas CP190/Chromator are responsible for the physical interactions required for long-range contacts (second layer). This network of organized, multi-layer interactions could explain the different activities of insulators as chromatin barriers, enhancer blockers, and transcriptional regulators, and suggest a general mechanism for how insulators may shape the organization of higher-order chromatin during cell division.

## Author Summary

Chromatin insulators mediate specific long-range DNA interactions required for the three dimensional organization of the interphase nucleus and for transcription regulation, but the mechanisms underlying the formation of these interactions is currently unknown. In this manuscript, we investigate the molecular associations between different protein components of insulators (BEAF32, CP190 and Chromator) by biochemical and biophysical means, and develop a novel biophysical assay to determine what factors are necessary and essential for the formation of long-range DNA interactions (LRI). Importantly, we show that CP190 and Chromator are able to mediate LRIs between specifically-bound BEAF32 nucleoprotein complexes. This ability of CP190 and Chromator to establish LRI requires specific contacts between BEAF32 and their C-terminal domains, and dimerization through their N-terminal domains. In particular, the BTB/POZ domains of CP190 form a strict homodimer. We propose a general model for insulator function in which BEAF32/dCTCF/Su(HW) provide DNA specificity, whereas CP190/Chromator are responsible for the physical interactions required for long-range contacts. This network of organized, multi-layer interactions could explain the different activities of insulators, and suggest a general mechanism for how insulators may shape the organization of higher-order chromatin during cell division.

## Introduction

The physical organization of eukaryotic chromosomes is key for a large number of cellular processes, including DNA replication, repair and transcription [1]–[6]. Chromatin insulators are genetic elements implicated in the organization of chromatin and the regulation of transcription by two independent modes of action: ‘enhancer blocking’ insulators (EB insulators) interfere with communications between regulatory elements and promoters, whereas ‘barrier’ insulators prevent the spread of silenced chromatin states into neighboring regions [7]–[9]. Recently, insulator elements have been implicated in chromosome architecture and transcription regulation through their predicted binding to thousands of sites genome-wide. For instance, insulators were shown to regulate transcription of distinct gene ontologies, to separate distinct epigenetic chromatin states, and to recruit H3K27me3 domains to Polycomb bodies [10]–[13].

In *Drosophila*, five insulator families have been identified, that differ by their DNA-binding protein (insulator binding protein, or IBP): Suppressor of Hairy-wing [Su(Hw)] [14], boundary element-associated factor (BEAF32) [15], Zeste-white 5 (Zw5) [16], the GAGA factor (GAF) [17], and dCTCF [18], a distant sequence homologue of mammalian CTCF. Two BEAF32 isoforms exist (BEAF32A and BEAF32B). In this paper, we will only consider BEAF32B (which will be referred to as BEAF32) as: (i) BEAF32B represents more than 95% of the binding peaks detected by chip-seq in cell lines [11], (ii) BEAF32A binding does not play a role in the insulating function of BEAF [19], and (iii) BEAF32A expression is not essential for the development of embryos in adult flies [20]. IBPs are often necessary but not sufficient to ensure insulation activity at a specific locus, and several insulator co-factors have been shown to be additionally required. Particularly, Centrosomal Protein 190 (CP190) [21], a protein originally described for its ability to bind to the centrosome during mitosis [22], was shown to play a crucial role in the insulation function of various IBPs [10], [23], [24].

Insulator proteins often associate in clusters of overlapping binding sites more often than would be expected by chance, suggesting that these factors often bind as a complex to the same genetic locus. For instance, BEAF32, dCTCF and CP190 binding sites most often cluster with at least another factor (∼70, ∼77 and >90%, respectively) [25]. In addition, insulators show a large compositional complexity, as demonstrated by the frequencies of binding of different combinations of insulator associated proteins: CP190 associates with its most common partner BEAF32 (∼50%), but also to a lesser extent to dCTCF and Su(HW) (25 and 20%, respectively), while BEAF32, dCTCF, and CP190 cluster together in >15% of CP190 binding sites [10], [25], [26]. This compositional complexity may be key to understanding the locus-specific functions of insulators.

A critical feature of Drosophila and vertebrate insulators is their ability to form specific long-range DNA interactions (hereafter LRIs) [27]–[34]. Three-dimensional loops have been implicated in all levels of chromatin organization ranging from kb-size loops to larger intra-chromosomal loops hundreds of kb in size [6], [35], [36]. To date, it is unclear what factors provide the physical interactions required for the formation and regulation of LRIs. In addition to binding the specific insulator sequences, IBPs have been proposed to be sufficient to bridge two distant DNA molecules [10], [37]. However, other factors such as CP190, Mod(mdg4), or cohesin have been implicated in the formation of LRIs [10], [38]–[40].

The observation that most CP190 binding sites co-localize with insulator binding proteins (>90%) [10], [25] prompted the hypothesis that CP190 is a common regulator of different insulator classes [10], [40]. CP190 is composed of a BTB (**b**ric-a-brac, **t**ramrack, and **b**road complex)/POZ (**po**xvirus and **z**inc-finger) domain, four predicted C_2_H_2_ zinc-finger motifs, and an E-rich, C-terminal region. Importantly, CP190 has been recently shown to preferentially mark chromatin domain barriers [13]. These barriers are also heavily bound by other insulator proteins, such as BEAF32, dCTCF and to a lesser extent Su(HW), and have been shown to often form LRIs [12]. Overall, these data suggest a role for CP190 in participating in the three dimensional folding of the genome by the formation of long-range interactions.

Surprisingly, a second factor, called Chromator, was also shown to be overrepresented at physical domain barriers [13]. During mitosis, Chromator forms a molecular spindle matrix with other nuclear-derived proteins (Skeletor and Megator) [41]. In contrast, during interphase Chromator localizes to inter-band regions of polytene chromosomes [42], [43] and plays a role in their structural regulation as well as in transcriptional regulation [44]. Chromator can be divided into two main domains, a C-terminal domain containing a nuclear localization signal, and an N-terminal domain containing a chromo-domain (ChD) required for proper localization to chromatin during interphase [45].

Here, we investigate the molecular associations between different components of insulator complexes (BEAF32, CP190 and Chromator) by biochemical and biophysical means. We developed a unique assay to determine what factors are necessary and essential for the formation of long-range DNA interactions, and show that BEAF32 is necessary but not sufficient to bridge long-range interactions. In contrast, addition of CP190 or Chromator is sufficient to mediate LRI between specifically-bound BEAF32 nucleoprotein complexes. This ability of CP190 and Chromator to establish LRI requires specific contacts between BEAF32 and their C-terminal domains, and dimerization through their N-terminal domains. In particular, the BTB/POZ domains of CP190 form a strict homodimer, and its C-terminal domain interacts with several IBPs. We propose a general model for insulator function in which BEAF32/dCTCF/Su(HW) provide DNA specificity (first layer proteins) whereas CP190/Chromator are responsible for the physical interactions required for long-range contacts (second layer). The multiplicity of interactions between insulator binding and associated proteins could thus explain the different activities of insulators as chromatin barriers, enhancer blockers, and transcriptional regulators.

## Results

### Quantitative DNA binding activity of BEAF32, CP190 and Chromator

**Figure 1:**
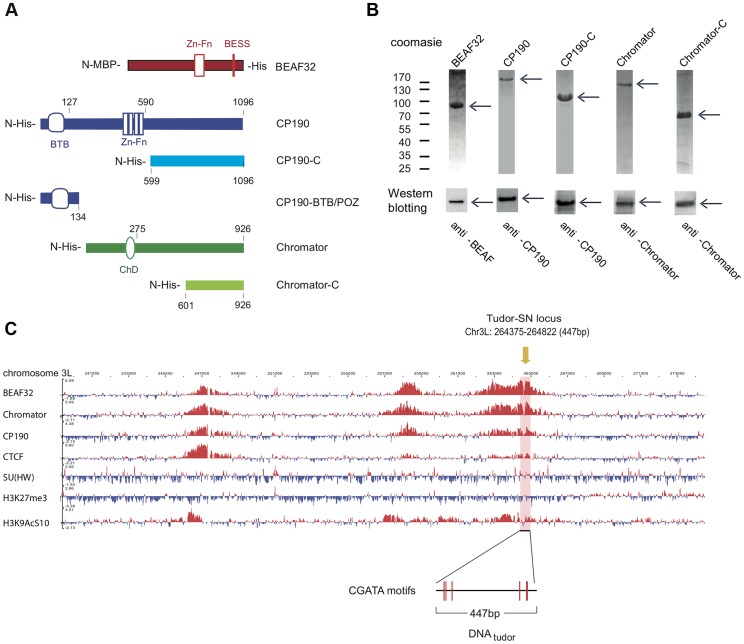
Protein constructs, protein purification, and genomic localization of insulator proteins and associated factors. (A) Description of protein constructs used in this study. C2H2 zinc-finger motifs (Zn-Fn) are shown as vertical rectangles, BESS motifs as a vertical red line, BTB/POZ domains as rounded boxes and chromo-domains (ChD) as ellipses. N-terminal domains (N-) are always on left. Lengths of each domain or fragment is indicated in number of amino-acids from the N-terminal end. His indicates a 6-Histidine tag, and MBP the maltose binding-protein. (B) Purity of purified BEAF32, CP190, CP190-C, Chromator, and Chromator-C was assessed by poly-acrylamide gel electrophoresis (PAGE, Coomassie blue staining, top panel), and resulted in single bands (>95% purity, see arrows). Molecular weight ladder is shown on the left. Western-blot analysis (bottom panel) of each purified protein shows the specific recognition by each of the antibodies developed. (C) Binding profile of insulator-associated proteins (BEAF32, Chromator, CP190, dCTCF, Su(HW)) and epigenetic marks (H4K27m3, and H3K9AcS10) in chromosome 3L from ModEncode data (S2 cells; Generic Genome Browser version 2.40). Tracks used are described in Supplementary Table S6. For each protein, the track depicts the MAT score of each probe plotted on the y-axis versus chromosomal position plotted along the x-axis. The genomic region used for EMSA-analysis (DNA_tudor_, part of the *Tudor-SN* lucus) is highlighted in pink (3L: 264375–264822). DNA_tudor_ contains six CGATA binding motifs.

BEAF32 co-localizes genome-wide with CP190 and Chromator, but the molecular mechanisms underlying this co-localization are unknown. To investigate whether this observed co-localization was due to direct protein-protein interactions, we heterologously expressed and purified BEAF32, CP190, Chromator and several protein subdomains. BEAF32 was expressed as a MBP (Maltose-Binding Protein) fusion protein (Figure 1A–B), since wild-type BEAF32 was mainly insoluble. CP190, Chromator, their C-terminal domains (CP190-C and Chromator-C, respectively), and CP190-BTB/POZ were heterologously expressed as His-tagged fusions (Figure 1A–B, and Materials and Methods). After purification, proteins were >95% pure and were specifically recognized by the corresponding antibodies (Figure 1B, and Materials and Methods).

**Figure 2:**
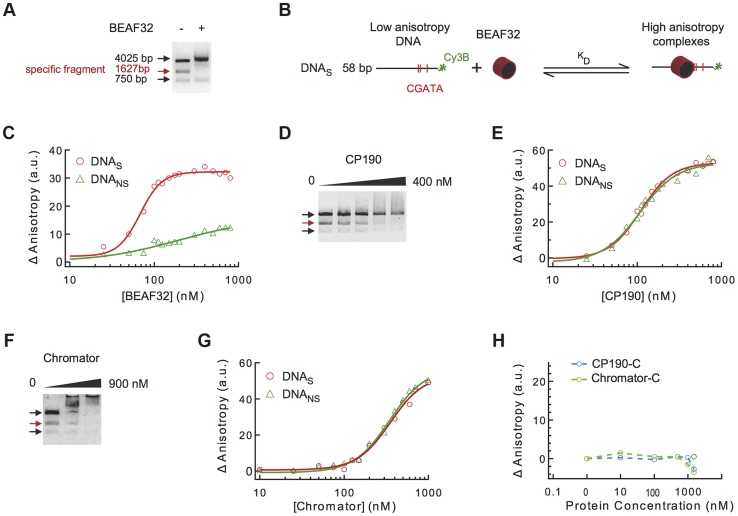
Binding of insulator factors to DNA. (A) Electric mobility shift assay (EMSA) of BEAF32-DNA_tudor_ complexes. A plasmid containing DNA_tudor_ was digested resulting in three linear fragments of size 750, 4025 and 1627 bp (the fragment containing DNA_tudor_, red). Addition of BEAF32 (200 nM) leads to the preferential disappearance of the band containing CGATA motifs. (B) Scheme representing the experimental setup for fluorescence anisotropy measurements of BEAF32-DNA binding equilibrium. Binding of BEAF32 to DNA_S_ (short DNA fragment containing three CGATA motifs) leads to an increase in the size of the complex that can be detected by an increase in the fluorescence anisotropy signal. K_D_ represents the apparent equilibrium dissociation constant of the complex. (C) BEAF32 binding isotherms for DNA_S_ (red circles) and DNA_NS_ (DNA fragment of the same size as DNA_S_ but with no CGATA motif, green triangles). Solid lines represent fits to a single-site binding (green) or a Hill model (red). (D) EMSA of CP190-DNA_tudor_ complexes show no specificity of DNA binding for CP190 at this genomic locus. In contrast to BEAF32, CP190 shifted the three DNA fragments with similar efficiency even at high protein concentrations (400 nM). Concentrations used were: 0, 100, 200, 300 and 400 nM, respectively. The decrease in the intensity of the top band is less pronounced due to intensity saturation. (E) CP190 binding isotherms for DNA_S_ (red circles) and DNA_NS_ (green triangles). Solid lines represent fits to a Hill model. CP190 binds both fragments with no specificity and equal affinity. (F) EMSA of Chromator-DNA_tudor_ complexes show no specific binding for Chromator at this genomic locus. Concentrations used were: 0, 450, and 900 nM, respectively. The intensity of all bands is decreased to the same extent by the binding of Chromator, reflecting non-specific binding to these DNA fragments. (G) Chromator binding isotherms for DNA_S_ (red circles) and DNA_NS_ (green triangles). Solid lines represent fits to a Hill model. Consistent with (F), Chromator binds both fragments with no specificity. (H) CP190-C (light blue) and Chromator-C (green) binding isotherms for DNA_S_. Addition of large protein concentration does not lead to detectable changes in fluorescence anisotropy.

A typical example of co-localization of these factors can be found at the *Tudor-SN* locus, a genomic region that shows a strong localization pattern for BEAF32, CP190, and Chromator but not for dCTCF or Su(HW) (Figure 1C), and contains six specific binding sites for BEAF32 (CGATA motifs) [19]. To directly test whether BEAF32 was able to specifically bind to this genomic site, we PCR-amplified a 447 bp DNA fragment from *Tudor-SN* that contained six CGATA motifs (hereafter DNA_tudor_, Figure 1C). First, we used an electric mobility shift assay (EMSA) in which a plasmid containing the DNA_tudor_ insertion was restricted and used as a substrate (Figure 2A). The restriction reaction produced three different DNA fragments of 750, 1627 and 4025 bp, the second of which contained the 447-bp DNA_tudor_ insertion, and was the only DNA fragment harboring specific CGATA motifs. The specific binding of factors to these different DNA fragments was assessed by quantifying the disappearance of unbound DNA species, as bound species often produced smeared bands due to rapid association/dissociation of proteins from DNA at low affinities and due to the low resolution of the gel matrix. The binding of BEAF to DNA was specific, as only the DNA_tudor_-containing band was preferentially shifted by addition of BEAF32 (Figure 2A).

Secondly, to quantify the affinity and specificity of DNA binding by BEAF32, we implemented a fluorescence anisotropy-based assay that directly measures the binding of proteins to DNA. The binding of proteins, such as BEAF32, to short fluorescently-labeled DNA fragments decreases the rotational diffusion of the DNA molecule and increases the fluorescence anisotropy of the attached fluorophore (Figure 2B) [46]. BEAF32 binds with a moderate apparent affinity to non-specific DNA (58 bp DNA fragment with no CGATA motif, hereafter DNA_NS_), and the binding isotherm can be well described by a simple single-site model (Eq.1, Text S1, *K*_D_ = 165±30 nM, Figure 2C). In contrast, BEAF32 binds to a specific DNA fragment of the same length (58 bp; DNA fragment containing three CGATA motifs from *Tudor-SN*, hereafter DNA_S_) with a higher affinity and displaying a degree of cooperativity (Figure 2C). The binding isotherm cannot be fitted by a single-site model, thus we turned to a Hill model (Eq. 2, Text S1) with a resulting apparent affinity of *K*_D_ = 68±5 nM and a Hill coefficient of *n* = 3±0.4. In addition, the change in fluorescence anisotropy signal was larger for DNA_S_ (32±2 anisotropy units) than for DNA_NS_ (12±5 anisotropy units), indicating that BEAF32-DNA_S_ makes a larger complex. Overall, these results indicate a cooperative binding of BEAF32 to CGATA motifs, suggesting oligomerization of BEAF32 at genomic sites containing multiple CGATA motifs. These results were consistent with competitive inhibition experiments (Supplementary Figure S1A). Equivalent fits of the DNA_NS_ binding isotherm to a Hill model produced a Hill coefficient of 0.9±0.3, consistent with cooperative binding of BEAF32 to DNA_S_ being due to the presence of CGATA motifs.

CP190 contains a BTB/POZ domain and is predicted to possess four classical C2H2 zinc-finger motifs that could be involved in direct DNA binding. It is unclear whether CP190 can directly associate to DNA, or rather relies on its binding to other factors to target specific binding sites [21], [47]. To address this question, we investigated the ability of CP190 to bind to *Tudor-SN*. This locus displays CP190 binding by Chip-chip [25], [48] (Figure 1C and Supplementary Table S6) and may thus contain moderate affinity sites for CP190. By EMSA, we observed that CP190 associated equally well to all DNA fragments, with no specificity shown for the DNA_tudor_-containing fragment (Figure 2D). Next, we tested the binding specificity of CP190 by fluorescence anisotropy, using two different dsDNA fragments (DNA_S_ and DNA_NS_). DNA_S_ should contain the potential CP190 moderate affinity sites giving rise to the *in vivo* binding of CP190 to *Tudor-SN*, while DNA_NS_ is a DNA fragment of the same length but with a random sequence serving as a control for specificity. In agreement with EMSA, fluorescence anisotropy experiments showed moderate DNA binding affinity but no specificity (*K*_D_ = 109±5 nM, *n* = 2±0.3 for CP190 on both DNA_S_ and DNA_NS_, Figure 2E). These results are supported by competition experiments (Supplementary Figure S1B), and are in agreement with similar experiments showing that CP190 fails to show any specificity when using a dsDNA fragment containing the predicted binding sequence of CP190 [25] (Supplementary Figure S6). Overall, these results are consistent with the specificity of *in vivo* binding of CP190 to *Tudor-SN* being mediated by other factors.

Next, we tested whether the C-terminal domain of CP190 was involved in the ability of CP190 to bind DNA non-specifically by determining the DNA binding properties of CP190-C, a protein construct that contains neither BTB/POZ nor the zinc-finger motif (Figure 1A). CP190-C was not able to bind DNA_S_ (Figure 2H), consistent with the non-specific association of CP190 to DNA being mediated by the N-terminal domain of CP190. Binding competition experiments of pre-bound BEAF32-DNA_S_ are inconsistent with CP190-BTB/POZ being involved in DNA binding (Supplementary Figure S1D), but further experiments will be required to determine the contribution of the different domains in the N-terminus of CP190 to DNA association. In addition, we cannot exclude the possibility that other factors or post-translational modifications may partially affect the mechanism of DNA binding by CP190. However, the ubiquitous co-localization of CP190 with factors displaying specific DNA-binding activities (BEAF32, dCTCF, Su(HW)) (>90%) [25] suggests that the presence of CP190 at specific loci is mediated in most cases by other proteins. From these experiments, we cannot exclude the possibility that CP190 may bind specifically to other genomic sites.

The ability of Chromator to associate to DNA has not been described so far, although its association to chromatin has been suggested to require its Chd-containing N-terminal domain (Yao et al, 2012). Despite the presence of high affinity *in vivo* sites for Chromator in *Tudor-SN*, our EMSA and fluorescence anisotropy experiments showed that Chromator binds DNA non-specifically (Figure 2F–G) and with a lower affinity than BEAF32 or CP190 (*K*_D_ = 360±30 nM and *n* = 2±0.2, see Figure 2G and Supplementary Figure S1C). Chromator-C did not present any DNA binding activity (Figure 2H), suggesting that Chromator binding to DNA requires its N-terminal domain or uncharacterized post-transcriptional modifications.

### BEAF32 forms a molecular complex with CP190 and Chromator

**Figure 3:**
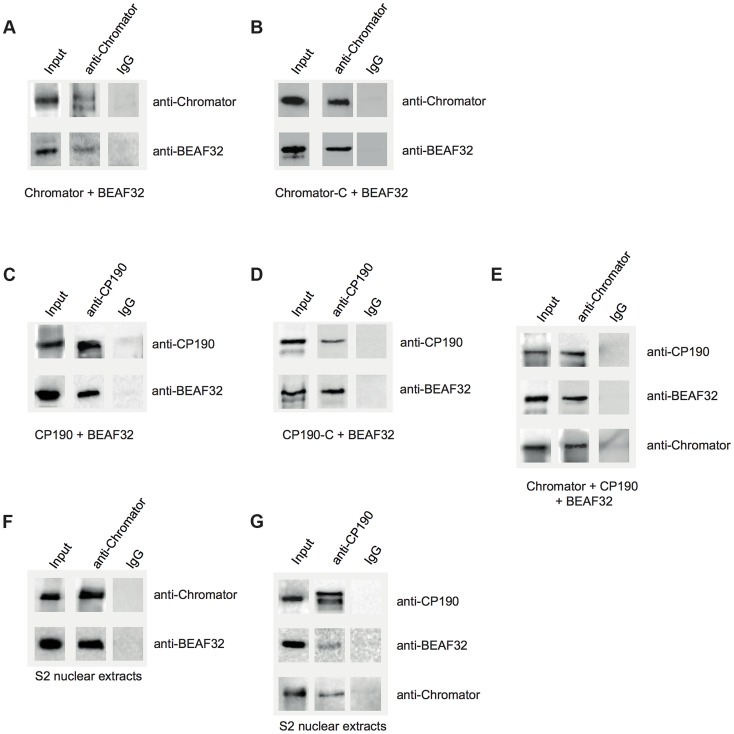
Interactions between insulator factors. (A) Co-immunoprecipitation pulldown assay (co-IP) with heterologously purified BEAF32 and Chromator. Goat-IgG or purified guinea-pig polyclonal antibodies against Chromator were covalently coupled to agarose beads. BEAF32 and Chromator were incubated and analyzed by SDS-PAGE followed by Western-Blot-analysis. Lane 1 (input) shows the presence of both BEAF32 and Chromator in the mix. Both proteins are retained by an anti-Chromator column, but not by an anti-goat-IgG column. (B) Co-IP of purified BEAF32 and Chromator-C. Chromator antibody recognizes Chromator and Chromator-C equally well (Materials and Methods). A mix of BEAF32/Chromator was incubated and analyzed by PAGE/Western blotting as before. Both BEAF32 and Chromator-C remain bound to an anti-Chromator column, consistent with the interaction between BEAF32 and Chromator being mediated by its C-terminal domain. (C) Co-IP of purified BEAF32 and CP190. A mix of BEAF32/CP190 was incubated and analyzed by PAGE/Western blotting. Both BEAF32 and CP190 remain bound to a rabbit anti-CP190 column, suggesting a direct interaction between these proteins. (D) BEAF32/CP190 interactions are mediated by CP190-C. A mix of BEAF32/CP190-C was incubated and analyzed by PAGE/Western blotting. Both BEAF32 and CP190-C remain bound to an anti-CP190 column, but not to the control anti-IgG column. (E) A mix of BEAF32, Chromator, and CP190 was incubated and analyzed by PAGE/Western blotting. The three proteins are bound to an anti-Chromator column, but not to the control anti-IgG column. (F) S2 nuclear extracts were incubated in an anti-Chromator or anti-IgG column and analyzed by PAGE/Western blotting. Both BEAF32 and Chromator remain bound to the anti-Chromator column, suggesting that these proteins interact in vivo. (G) S2 nuclear extracts were incubated in an anti-CP190 or anti-IgG column and analyzed by PAGE/Western blotting. Consistent with previous results, BEAF32, CP190 and Chromator remain bound to the anti-CP190 column, suggesting that these proteins are part of the same complex in vivo.

Next, we investigated whether BEAF32 directly interacts with CP190 and Chromator by using several complementary approaches. First, we employed co-immunoprecipitation (co-IP) to detect protein-protein interactions with heterologously purified proteins. A guinea pig-anti-Chromator-antibody was covalently linked to a column and a mix of purified BEAF32 and Chromator were incubated in the column for 60 min, eluted and analyzed by western blotting (Figure 3A, see full bands of all co-IPs in Supplementary Fig. S2C–I). Western-Blot analysis of the elution clearly showed the specific interaction between BEAF32 and Chromator (Figure 3A, middle column), whereas neither BEAF32 nor Chromator were found to bind to an IgG-antibody column (Figure 3A, right column). Importantly, BEAF32 did not bind to an anti-Chromator column in the absence of Chromator (Supplementary Figure S2B). To investigate what domain of Chromator is involved in interactions with BEAF32, we performed co-IP experiments in which a mix of BEAF32 and Chromator-C were incubated in a column covalently bound by antiChromator antibody, and the elution analyzed by western blotting. Interestingly, BEAF32 is specifically retained in the Chromator-C column, consistent with BEAF32/Chromator interactions being mediated by the C-terminal domain of Chromator (Figure 3B). Additionally, Chromator is retained in a CP190 column, an interaction that seems to be specifically mediated by CP190-BTB/POZ (Supplementary Figure S2K).

Similar co-IP experiments were performed to test putative BEAF32-CP190 interactions. A rabbit-anti-CP190-antibody was covalently linked to a resin and incubated with a purified mix of BEAF32 and CP190 or CP190-C. BEAF32 binds efficiently to both CP190 and CP190-C (Figure 3C–D), but failed to interact with CP190-BTB/POZ (Supplementary Figure S7). BEAF32 is not recognized by CP190 antibodies and was not retained by an anti-CP190 column (Supplementary Figures S2A–B). These results indicate that BEAF32/CP190 interactions are mediated by the C-terminal domain of CP190, although we cannot discard an additional contribution of the zinc-finger domains of CP190 to this interaction. Interestingly, both BEAF32 and CP190 were retained in an anti-Chromator antibody column (Figure 3E), consistent with binary interactions between BEAF32 and CP190/Chromator and with interactions between CP190 and Chromator.

To test whether these interactions are physiologically relevant, we performed Co-IP experiments using S2 nuclear extracts (see Materials and Methods). Interactions between BEAF32, Chromator and CP190 were clearly detected while either using anti-Chromator or anti-CP190 (Figures 3F–G, respectively) antibodies. Overall, these results suggest that BEAF32, Chromator and CP190 are part of the same molecular complex. However, further work is necessary to determine the architecture and stoichiometry of this complex.

### BEAF32 requires either CP190 or Chromator to form higher-order DNA interactions

**Figure 4:**
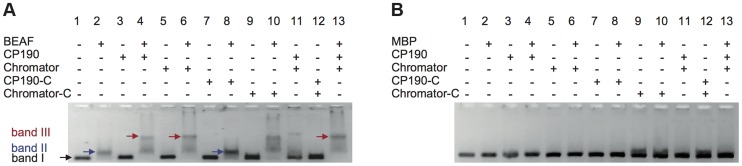
Insulator factors interact upon DNA binding. (A) Native agarose band-shift assay of BEAF32-DNA_tudor_, and higher order complexes. Lane 1, 476 bp DNA_tudor_; lane 2, DNA_tudor_ incubated with BEAF32; lane 3, DNA_tudor_ incubated with CP190; lane 4, DNA_tudor_ incubated with BEAF32 and CP190; lane 5, DNA_tudor_ incubated with Chromator; lane 6, DNA_tudor_ incubated with BEAF32 and Chromator; lane 7, DNA_tudor_ incubated with CP190-C; lane 8, DNA_tudor_ incubated with BEAF32 and CP190-C; lane 9, DNA_tudor_ incubated with Chromator-C; lane 10, DNA_tudor_ incubated with BEAF32 and Chromator-C; lane 11, DNA_tudor_ incubated with CP190 and Chromator; lane 12, DNA_tudor_ incubated with CP190-C and Chromator-C; lane 13, DNA_tudor_ incubated with BEAF32, CP190 and Chromator. Band 1 represents DNA_tudor_. Band 2 represents the complex between BEAF32 and DNA_tudor_. Band 3 represents the BEAF32/DNA_tudor_ complex super-shifted by binding of CP190, Chromator, Chromator-C, or the addition of both CP190 and Chromator. The shift of band 1 requires the presence of BEAF32. Protein concentrations used: BEAF32 (400 nM), CP190 (50 nM), CP190-C (50 nM), Chromator (100 nM), Chromator-C (100 nM). (B) Native agarose band-shift assay of MBP-DNA_tudor_. This experiment used the same protein mixes and concentrations as those used in (A) but replacing BEAF32 by MBP. No shifted band is apparent.

Next, we investigated whether interactions among BEAF32, CP190 and Chromator lead to the formation of higher-order DNA interactions. First, we used EMSA to test whether BEAF32-CP190/Chromator sub-complexes bind to the 447 bp DNA_tudor_ fragment (Figure 1C). BEAF32 binding to DNA_tudor_ (Figure 4A, Lane 1, band I) produced a discrete shift corresponding to a BEAF32/DNA_tudor_ complex (Figure 4A, lane 2, band II). Consistent with previous results, neither CP190/Chromator (as the concentrations used here were lower than the K_D_), nor their C-terminal fragments were able to bind DNA_tudor_ under these conditions (Figure 4A, band I, lanes 3, 5, 7 and 9, respectively). Interestingly, a second band with lower electrophoretic mobility appeared only when BEAF32 and either CP190 or Chromator were simultaneously present (Figure 4A, lanes 4 and 6, band III). Furthermore, this complex did not form when CP190 was replaced by CP190-C (Figure 4A, compare lanes 4 and 8), and exhibited a similar intensity when Chromator-C was used instead of full-length Chromator (lane 10 in Figure 4A). Control experiments where BEAF32 was replaced by MBP showed that the formation of the protein-DNA complexes leading to bands II and III required the presence of BEAF32 (Figure 4B). Band II thus corresponds to a complex formed by BEAF32 and DNA_tudor_, while band III indicates the presence of specific interactions between DNA-bound BEAF32 and CP190, Chromator, and Chromator-C.

**Figure 5:**
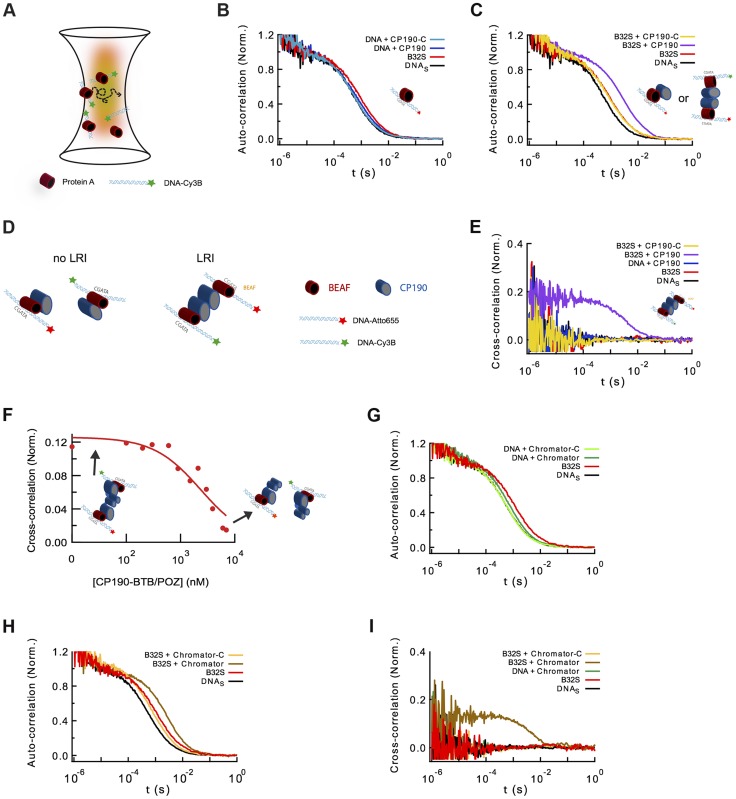
Formation of long-range interactions by insulator proteins. (A) Scheme depicting a typical fluorescence fluctuation spectroscopy configuration. Fluorescently-labeled dsDNA fragments (cyan ribbon with green star) diffuse in and out of an excitation volume (red gradient) producing a time-dependent fluctuation in the fluorescence signal. Binding of protein (red cylinder) to DNA lead to a larger molecular complex, with a corresponding increase in its diffusion time. (B) Normalized auto-correlation of DNA_S_-Cy3B (2.5 nM, black), and shift in the auto-correlation curve due to BEAF32 binding (400 nM, B32S complex, red) or CP190 binding (50 nM, blue). No noticeable change in the diffusion time is observed when adding CP190-C (50 nM, light blue) to DNA_S_-Cy3B. (C) Normalized auto-correlation of DNA_S_-Cy3B (black), B32S (red), and a complex of B32S with CP190 (violet) or CP190-C (yellow). Addition of CP190 (50 nM) to B32S (400 nM) considerably increased the diffusion time, consistent with direct BEAF32/CP190 interactions leading to the formation of a higher molecular mass complex. Inset shows the two possible models that could lead to this increase in diffusion time. (D) Scheme presenting the two models tested by fluorescence cross-correlation spectroscopy. The formation of long-range interactions between B32S complexes (with either a Cy3B- or an atto655-labeled DNA_S_ fragment) lead to a cross-correlation signal between these two colors. In contrast, the absence of cross-correlation signal implies no long-range interaction between B32S complexes. BEAF is shown in red, and CP190 in blue. DNA_S_ is represented by a cyan ribbon with a star representing the fluorophore at its 5′-end. (E) Cross-correlation between the two fluorophores was only observed in the presence of CP190 (50 nM) and B32S (400 nM), and not when DNA alone, CP190+DNA_S_, or CP190-C+B32S were used at the same concentrations. (F) CP190 (50 nM) was pre-incubated with B32S (400 nM BEAF32), leading to a complex with a large cross-correlation signal in which CP190 forms long-range contacts between CGATA motifs (see inset scheme). The titration of this complex with CP190-BTB/POZ leads to the disappearance of the cross-correlation signal, consistent with the CP190-BTB/POZ domain being responsible for the CP190-CP190 interactions required for establishing long-range interactions. (G) Normalized auto-correlation of DNA_S_-Cy3B (2.5 nM, black), and shift in the auto-correlation curve due to BEAF32 binding (800 nM, B32S complex, red) or Chromator binding (100 nM, green). No noticeable change in the diffusion time is observed when adding Chromator -C (100 nM, light green) to DNA_S_-Cy3B. (H) Normalized auto-correlation of DNA_S_-Cy3B (black), B32S (red), and a complex of B32S with Chromator (dark yellow) or Chromator-C (yellow). Addition of Chromator (100 nM) to B32S (800 nM) considerably increased the diffusion time, consistent with direct BEAF32/Chromator interactions leading to the formation of a higher molecular mass complex. The small decrease in diffusion time observed upon addition of Chromator-C to B32S was not due to BEAF32 dissociating from DNA (Supplementary Figure S4), but probably due to a change in the translational diffusion of the complex triggered by a rearrangement of BEAF32 on DNA_S_ upon interaction with Chromator-C [90], [91]. (I) Cross-correlation signal was only observed in the presence of Chromator (100 nM) and B32S (800 nM BEAF32), but not when DNA alone, Chromator+DNA_S_, or Chromator-C+B32S were used at the same concentrations.

To characterize the different complexes formed by BEAF32, CP190 and Chromator, we turned to fluorescence correlation spectroscopy (FCS). FCS uses the fluctuations in the number of freely diffusing fluorescently-labeled molecules within a confocal volume to characterize their diffusion time [49], [50] (Figure 5A). Thus, the formation of protein-DNA complexes can be monitored by the increase in the apparent size (related to the diffusion time) of a fluorescently-labeled dsDNA fragment upon protein binding. In our case, we used the 58 bp DNA_S_ fragment (harboring three CGATA motifs, 5′-Cy3B labeled, see Materials and Methods) as a fluorescent reporter to quantitatively monitor the formation of BEAF32/CP190/Chromator complexes (Figure 5D). Identical results were obtained with an atto655-DNA_S_ probe (Supplementary Figure S3). Incubation of DNA_S_ with saturating concentrations of BEAF32 (400 nM) led to an increase in its apparent diffusion time from 0.53±0.03 to 0.85±0.04 ms, obtained by fitting our measurements to a 3-D diffusion model with a triplet state (Eq. 3, Text S1). This shift is consistent with the binding of BEAF32 to DNA_S_ leading to the production of a molecular complex (hereafter B32S complex) with an increased apparent size (Figure 5B). The addition of low concentrations of CP190 (50 nM) to DNA_S_ produced a small increase in the diffusion time (from 0.53±0.03 to 0.65±0.09 ms), consistent with the sub-affinity concentrations used. In contrast, CP190-C did not change the diffusion time of DNA_S_ (Figure 5B), in agreement with our previous results showing no DNA-binding activity for this domain of CP190 (Figure 2H).

Next, we investigated whether CP190 binds to B32S complexes. We observed that the incubation of pre-formed B32S complexes with low-concentrations of CP190 (50 nM) led to a considerable increase in the size of complexes (Figure 5C). This low CP190 concentration (below its affinity) was used to enhance the specificity of CP190/BEAF32 interactions and limit the direct binding of CP190 to DNA_S_. Conversely, the addition of CP190-C to B32S slightly decreased the apparent size of the complex (Figure 5C). To ensure that this small decrease in diffusion time was not due to the dissociation of BEAF32 from DNA_S_, we performed fluorescence anisotropy experiments. The anisotropy of pre-formed B32S complexes was independent of the concentration of CP190-C, but decreased to the anisotropy of free DNA_S_ upon addition of high salt concentrations (Supplementary Figure S4). These results indicate that the decrease in diffusion time observed in B32S/CP190-C complexes is not due to the dissociation of BEAF32 from DNA_S_, but to the change in the shape of the complex upon CP190-C binding. Overall, these results are consistent with either CP190 binding a B32S complex or triggering long-range inter-segment interactions between two B32S complexes.

To discriminate between these two models, we turned to fluorescence cross-correlation spectroscopy (FCCS). FCCS measures the correlated fluorescence intensity fluctuations of two spectrally-distinct, fluorescently-labeled molecules to quantitatively determine whether they are in the same molecular complex [50], [51]. When two DNA fragments labeled with different colors are part of the same molecular complex, their fluorescence fluctuations will be correlated (LRI), whereas no cross-correlation will be observed if the diffusion of the two DNA fragments is independent (no LRI, Figure 5D). We used a 50/50 mixture of DNA_S_ labeled with Cy3B and atto655. Since these two fluorophores can display a significant level of crosstalk between detection channels, introducing apparent cross-correlation in the absence of interaction, we used pulsed interleaved excitation (PIE-FCCS) [52], [53] a technique that eliminates this artifactual effect and allows quantitative fluorescence cross-correlation measurements. The cross-correlation signals were measured for DNA_S_, B32S, and solutions of pre-formed B32S complex incubated with either CP190 or CP190-C and fitted with Eq. 4 (Text S1). Neither DNA_S_, nor B32S showed cross-correlation (Figure 5E), demonstrating the inability of BEAF32 alone to mediate long-range intermolecular interactions between CGATA motifs. In agreement with our previous observations (lack of band III in lane 8, Figure 4A), addition of CP190-C to B32S did not trigger the formation of intermolecular complexes, suggesting that the E-rich domain of CP190 is not sufficient to generate LRIs *in vitro*. In contrast, these complexes were formed in the presence of full-length CP190, demonstrated by the appearance of a clear cross-correlation signal (18±6%, Figure 5E). From the DNA labeling efficiencies of Cy3B- and atto655-labeled oligonucleotides (∼57 and 97%, respectively), and the fact that a maximum of 50% of the bridged DNA can be observed in the cross-correlation amplitude (since Cy3B-Cy3B or atto655-atto655 complexes do not produce a cross-correlation signal), we can conclude that 65±22% of the B32S-atto655 complexes take part in LRIs mediated by CP190. Importantly, under these conditions CP190 alone was not able to generate LRIs (Figure 5E), and addition of neither full-length CP190 nor CP190-C affected the specific binding of BEAF32 to DNA_S_ (Supplementary Figure S4).

Thus, while CP190-C interacts with BEAF32, the N-terminal domain of CP190 appears necessary for the formation of inter-segment LRIs mediated by BEAF32-bound DNA *in vitro* (as CP190-C is not sufficient to mediate these interactions). In agreement with this model, the competition of pre-formed B32S-CP190-B32S complexes with the purified, isolated CP190-BTB/POZ domain (Figure 1A) led to the disappearance of cross-correlation signal (Figure 5F), but not to the displacement of BEAF32 from DNA_S_ (Supplementary Figure S1D).

### Structure of the CP190-BTB/POZ domain

Overall, the FCCS data strongly suggest that the BTB/POZ domain of CP190 is involved in the direct protein-protein interactions required for the establishment of long-range contacts. To directly test this hypothesis, we solved the crystal structure of the CP190-BTB/POZ. BTB/POZ motifs are widespread in eukaryotes (350 BTB/POZ-containing proteins in the human genome). Despite a low degree of primary sequence conservation (as low as 10%), the various structures reported in the literature are very similar (root mean square deviation, or RMSD ∼1–2 Å) with the overall architecture being composed of a cluster of five alpha helices capped on one end by three beta sheets. BTB/POZ motifs have been found to homodimerize, heterodimerize, and in rare cases to promote tetramerization. These different types of oligomerization states depend primarily on the surface residues involved in oligomerization and have been well documented elsewhere [54], [55].

**Figure 6:**
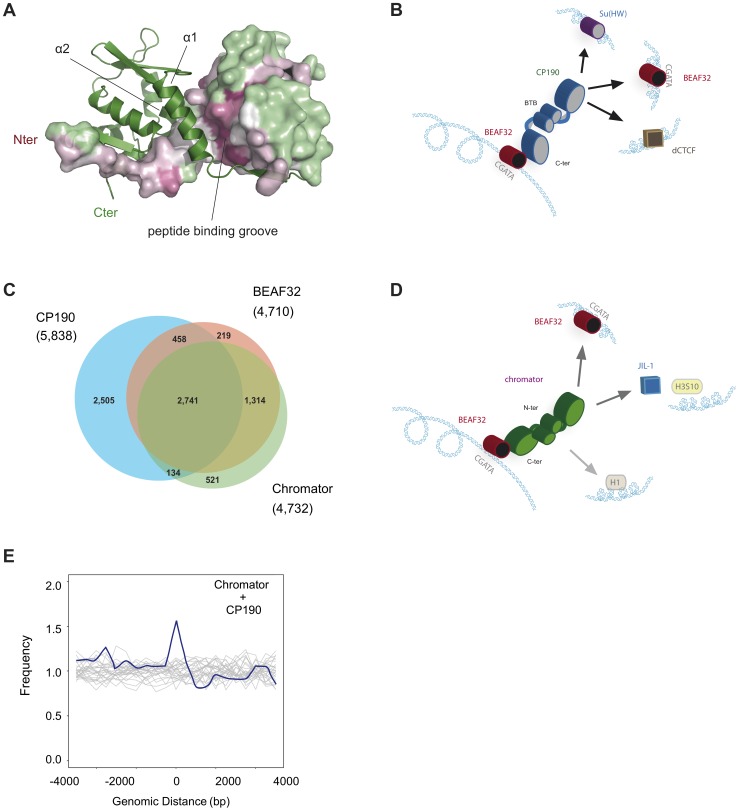
Structure of CP190-BTB/POZ, genome-wide localization of insulator factors at long-range contacts, and models. (A) CP190-BTB/POZ crystallizes as a homo-dimer. The secondary structure of one monomer is shown in green and the surface of the second monomer is color-coded by conservation (pink: high, green: low). Monomers are mainly held together by interactions between helices α1 and α2. The peptide binding groove and the N-terminal domains are highly conserved and may participate in protein-protein interactions (discussed in text). (B) Schematic model for the formation of long-range interactions by CP190. The BTB/POZ domains of CP190 (blue) interact to form a dimer. Contacts between the C-terminal domain of CP190 and BEAF32 (red) or other insulator binding proteins (Su(HW), blue, dCTCF, brown) can lead to the formation of hybrid long-range contacts. (C) Venn diagram showing the genome-wide overlap between BEAF32, CP190 and Chromator in S2 cells calculated from publicly available modENCODE ChIP-chip data. (D) Schematic model highlighting the possible roles of Chromator/BEAF32 interactions. Chromator (green) could act as a LRI-forming protein bridging BEAF32 (red) binding sites, as well as serve to recruit the JIL-1 kinase (blue box) to regions of active chromatin. (E) Aggregation analysis was performed on Hi-C data to identify proximity correlations and functional relationships between anchors (BEAF32 binding sites) and signals (CP190/Chromator binding sites). Aggregation profiles are built by aligning and aggregating the Hi-C signals of CP190/Chromator peaks at a certain genomic distance *d* (15<*d*<60 kbp) from BEAF32 binding sites. BEAF32 is used as the anchor and CP190/Chromator signals are aligned (at *d* = 0) and summed together. The y-axis shows the number of interactions every 500 bp normalized by the total number of sites (interacting and not interacting) that localize at the same distances from the anchor. Normalized aggregation Hi-C profiles for CP190 + Chromator are shown as blue solid lines, whereas control regions with no anchor are shown in grey.

The CP190-BTB/POZ domain crystallized as a stable and symmetric homodimer, in agreement with gel-filtration analysis. The overall structure is similar to classic BTB/POZ-ZF transcriptional factors where the N-terminal BTB/POZ domain is followed by several Zinc-Fingers domains (Figure 6A, Materials and Methods and Supplementary Table S1). The dimerization interface is stabilized by a swapped β-strand that forms a long groove where extended polypeptidic segments can bind in order to recruit other protein partners. The dimer interface (1902 Å^2^/monomer according to PISA 1.47 [56]) is composed of numerous hydrophobic interactions mainly from alpha helices α1 and α2 (i.e. W12, F15, F16, F23, L47…). The native homodimeric organization is also reinforced by the N-terminal strand (residues Glu2 to Asp10) being swapped: the β1 stand of a monomer interacts with the β5 strand of the other monomer. The sequence conservation among CP190 orthologs from insects (55 sequences analyzed using CONSURF [57]) show little conservation besides the domain core, the dimerization interface and the peptide binding groove (Figure 6A). Interestingly, this suggests that CP190-BTB/POZ does not form higher order macromolecular assemblies by itself while partner recruitment requires homo-dimerization. Importantly, we found that CP190-BTB/POZ forms strict homo-dimers (Figure 6A), consistent with the ability of CP190 to form LRIs.

### Role of Chromator in establishing long-range interactions

Finally, we used FCS and PIE-FCCS to test whether Chromator was able to mediate LRIs between two B32S complexes *in vitro*. The addition of 100 nM Chromator to DNA_S_ generated a small but noticeable change in the diffusion time (0.53±0.03 to 0.59±0.03 ms) (Figure 5G), consistent with our previous results (Figures 2F and 2G) indicating that Chromator interacts non-specifically with DNA. In contrast, incubation of DNA_S_ with Chromator-C did not induce any change in the diffusion time of the probe (Figure 5G), in agreement with anisotropy experiments (Figure 2H). Interestingly, addition of Chromator (but not of Chromator-C) to B32S considerably changed the diffusion time of the complex (Figure 5H), suggesting an interaction between Chromator and the B32S complex. Similarly to the results obtained for CP190, addition of Chromator to pre-formed B32S complexes led to a cross-correlation amplitude of 13±4%, corresponding to a total of 47±14% of the B32S-atto655 complexes bridged by Chromator interactions (Figure 5I). The formation of these complexes was not observed when Chromator-C was added to B32S, nor when Chromator was added to DNA_S_ in the absence of BEAF32 (Figure 5I). Overall, these results are consistent with interactions between the N-terminal domains of Chromator being required for the bridging function of Chromator, with Chromator-C providing the main direct interactions to BEAF32. We cannot discard, however, the possibility that Chromator-N may also partially interact with BEAF32.

## Discussion

Chromatin insulators promote higher-order nuclear organization through the establishment and maintenance of distinct transcriptional domains. Notably, this activity requires the formation of barriers between chromatin domains and the establishment of specific LRIs. In this paper, we investigated the molecular mechanism by which insulator proteins bind DNA, interact with each other and form long-range contacts.

### CP190 is responsible for the establishment of long-range contacts *in vitro*

Recently, genome-wide approaches have been used to investigate the roles of different insulator types in genome organization. Insulators enriched in both BEAF32 and CP190 are implicated in the segregation of differentially expressed genes and in delimiting the boundaries of silenced chromatin [25]. Notably, BEAF32 and CP190 are often found to bind jointly to the same genetic locus (>50% of CP190 binding sites contain BEAF32) [10], [25]. However, the molecular origin of this genome-wide co-localization was unknown as there was no direct proof of interaction between these proteins. Here, we showed, for the first time to our knowledge, that BEAF32 is able to interact specifically with CP190 *in vitro* and *in vivo*. In particular, we observed that this interaction is mediated by the C-terminal domain of CP190, with no implication of the C2H2 zinc-finger or the BTB/POZ domains, consistent with previous studies showing that the N-terminus of CP190 was not essential for its association with BEAF32 *in vivo* [58]. BEAF32 interacts specifically and cooperatively with DNA fragments containing CGATA motifs, consistent with previous observations [19]. In contrast, the binding of CP190 to DNA showed lower affinity and no specificity and required its N-terminal domain (containing four C2H2 zinc-fingers). Overall, these data suggest that one pathway for CP190 recruitment to DNA genome-wide requires specific interactions of its C-terminal domain with BEAF32. Other factors, such as GAF [38], are likely also involved in the recruitment of CP190 to chromatin, explaining why RNAi depletion of BEAF32 does not lead to the dissociation of CP190 from an insulator binding class containing high quantities of BEAF32 and CP190 [47]. We cannot discard that post-translational modifications in CP190 may also allow it to bind DNA directly and specifically, providing a second pathway for locus-specific localization.

In addition to acting as chromatin barriers, insulators have been typically characterized for their ability to block interactions between enhancers and promoters through the formation of long-range contacts [7], [16], [27], [28], [59]–[62]. Here, we developed a fluorescence cross correlation-based assay that allowed us, for the first time to our knowledge, to investigate the ability of BEAF32, CP190 and their complex to bridge specific DNA fragments, mimicking LRIs. We show that specific LRI can be stably formed between two DNA fragments containing BEAF32 binding sites, solely in the presence of both BEAF32 and CP190. Interestingly, LRI are displaced by competition *in trans* with the BTB/POZ domain of CP190, and LRIs are not observed in the presence of BEAF32 and CP190-C. Thus, both protein domains are required for the bridging activity of CP190. These data strongly suggest that the C-terminal domain is responsible for BEAF32-specific contacts whereas the N-terminal domain of CP190 is involved in the formation of LRI through CP190/CP190 contacts (Figure 6B). The role of the N-terminal domain of CP190 in protein-protein interactions is consistent with previous studies showing that N-terminal fragments of CP190 containing the BTB/POZ domains co-localize with full-length CP190 in polytene chromosomes [58].

BTB/POZ are a family of protein-protein interaction motifs conserved from Drosophila to mammals, and present in a variety of transcriptional regulators. BTB/POZ are found primarily at the N-terminus of proteins containing C2H2 zinc-finger motifs [63]–[65], and can be monomeric, dimeric, or multimeric [54], [55]. In fact, a recent study proposed that isolated CP190-BTB/POZ domains can exist as dimers or tetramers in solution [66]. The oligomerization behavior of CP190-BTB/POZ could have important implications for the role and mechanism by which CP190 bridges LRIs. Here, we showed that the BTB/POZ domains of CP190 forms homo-dimers with a large, conserved interaction surface (Figure 6A), consistent with these domains being responsible for the formation of the direct protein-protein interactions required for the establishment of long-range contacts. Interestingly, the oligomerization of CP190-BTB/POZ into homo-dimers implies a binary interaction between two distant DNA sequences, imposing important constraints for the mechanisms of DNA bridging by CP190.

In addition to interacting with BEAF32, CP190 is able to directly interact with other insulator binding proteins, such as dCTCF, Su(HW), and Mod(Mdg4) [21], [39], [66]–[68], or with the RNA interference machinery [69]. These interactions are usually mediated by the C-terminal domain of CP190, but a role for the C2H2 zinc-finger or the BTB/POZ domains in providing specific protein-protein contacts cannot be discarded [70]. In fact, an interesting feature of several homo-dimeric BTB/POZ domains is their ability to recruit a multitude of protein partners using a single protein-protein binding interface. For instance, several transcriptional co-repressors (BCOR, SMRT and NCor) are able to bind with micromolar affinity (2∶2 stoichiometry) to the BTB/POZ domain of BCL6, despite their low sequence homology [71], [72]. In this case, the mechanism of binding involves the formation of a third strand by the N-terminus of co-repressors folding onto the two strands exchanged by the BCL6-BTB/POZ monomers on their interface, with the rest of the minimal domain of interaction (10 residues) winding up along the lateral groove of the BCL6-BTB/POZ dimer (peptide binding groove in Figure 6A). In the case of CP190, the sequence and structural features of the conserved peptide binding groove within insect CP190-BTB/POZ domains suggest that the dimer interface of CP190 may act as a protein-protein interaction platform. Thus, the ability of BTB/POZ domains to form dimers and the promiscuous binding of CP190 to different insulator binding proteins (Su(HW), dCTCF [39], [67], and BEAF32) suggest not only that insulators share protein components [73], but also that CP190 may bridge long-range contacts involving distinct factors at each end of the DNA loop (Figure 6B). This model is consistent with previous proposals [73], and with the requirement of both C- and N-terminal domains of CP190 for fly viability [58]. Importantly, it provides a rationale for CP190 being a common factor between insulator binding proteins.

CP190 frequently binds with additional insulator binding proteins (∼85%), with BEAF32 and dCTCF being the most common partners (∼50% and ∼25%, respectively), and Su(Hw) amongst the least frequent partner (∼20%) [10], [25]. Importantly, BEAF32 does not show clustering with either dCTCF or Su(HW) in the absence of CP190 (<0.5% or ∼0.1%, respectively) [10], suggesting that the clustering of two insulator binding proteins requires CP190. The ability of CP190 to mediate LRIs between sites harboring different insulator binding proteins raises important questions: Are these LRIs specific? How is this specificity regulated? Are other factors or post-translational modifications involved in this selectivity? Future research will be needed to address these important questions.

### Interactions between BEAF32 and Chromator may lead to chromatin opening

Chromator localizes to inter-band regions of polytene chromosomes [42], [43] and binds to the barriers of physical domains genome-wide [13], however the mechanism leading to these localization patterns has been lacking. Previous studies showed that BEAF32 and Chromator co-localize at some genomic sites, and suggested that these proteins may participate in the formation of a single complex [@Gan:2011hy]. Here, we showed for the first time that BEAF32 directly and specifically interacts with Chromator *in vivo* and *in vitro*. This interaction is mediated by the C-terminal domain of Chromator, thus the ChD domain does not seem to be directly involved in interactions with BEAF32. Our results show that Chromator possesses a reduced affinity for DNA and binds with no sequence specificity to loci displaying strong Chromator binding peaks at the site tested (*Tudor-SN* locus, Figures 2G and 1C). Thus, we suggest that specific interactions between BEAF32 and Chromator may be responsible for its recruitment to polytene inter-band regions and domain barriers. Significantly, most BEAF32 binding sites genome-wide (>90%, Figure 6C and Supplementary Figure S5A) contain Chromator, suggesting an almost ubiquitous interaction between the two factors.

Interestingly, Chromator also co-localizes with the JIL-1 kinase at polytene inter-band regions and the two proteins directly interact by their C-terminal domains [41]. JIL-1 is an ubiquitous tandem kinase essential for Drosophila development and key in defining de-condensed domains of larval polytene chromosomes. Importantly, JIL-1 participates in a complex histone modification network that characterizes active, de-condensed chromatin, and is thought to reinforce the status of active chromatin through the phosphorylation of histone H3 at serine 10 (H3S10) [74]–[76]. Thus, BEAF32 could be responsible for the recruitment of the Chromator/JIL-1 complex to active chromatin domains to prevent heterochromatin spreading (Figure 6D) [@Gan:2011hy]. This mechanism would be consistent with the observation that BEAF32 localizes primarily to de-condensed chromatin regions in polytene chromosomes [15], is implicated in the regulation of active genes [10], [11], [25], [77] and delimits the boundaries of chromatin silencing [25].

### The different functional layers of chromatin insulators

CP190 is a common partner of BEAF32, dCTCF, and Su(HW), and has been thus proposed to play a role in the formation of long-range interactions at these insulators [10], [67]. On the other hand, both CP190 and Chromator have been recently shown to be massively overrepresented at barriers between transcriptional domains [12], [13]. In this paper, we show, for the first time, that only when CP190 or Chromator are present can long-range interactions between BEAF32-bound DNA molecules be generated. We provide strong evidence that the formation of *in vitro* LRI requires three ingredients: (1) binding of BEAF32 to its specific DNA binding sites; (2) specific interactions between the C-terminal domains of CP190/Chromator and BEAF32; and (3) homo- interactions between CP190/Chromator molecules mediated by their N-terminal ends.

To further investigate the roles of CP190 and Chromator in the formation of LRIs, we aggregated together statistically relevant contacts containing specific combinations of insulator factors from Hi-C data from embryos [13] (Figure 6E, and Materials and Methods). This analysis shows a relatively high correlation between the presence of BEAF32 and both CP190 and Chromator in sites displaying a high proportion of interacting bins between distant BEAF32 sites (Figure 6E), as compared with neighboring sites (16.9% of interacting bins for Chromator and CP190 sites; Wilcoxon test: p-value ∼1e-7). Thus, CP190 and Chromator may play a role at a subset of genetic loci by mediating and/or stabilizing interactions between BEAF32 and a distant locus bound by BEAF32 or a different insulator binding protein. Interestingly, the binding of BEAF32 to CGATA sites as multimers, and the existence of CP190-Chromator interactions suggest that long-range interactions at a single locus could involve hybrid/mixed complexes comprising at least these three factors.

These observations suggest a general model for insulator function in which BEAF32/dCTCF/Su(HW) provide DNA specificity (first layer proteins) whereas CP190/Chromator are responsible for the physical interactions required for long-range contacts (second layer). Direct or indirect interactions of first layer insulator proteins with additional factors (e.g. JIL-1, NELF, mediator) are very likely involved in directing alternative activities (e.g. histone modifications, regulation of RNAPII pausing) to specific chromatin loci. This model provides a rationale for the compositional complexity of insulator sequences [25] and for the multiplicity of functions often attributed to insulators (e.g. enhancer blocker, chromatin barrier, transcriptional regulator). Ultimately, a characterization of the locus-specific composition of insulator complexes and their locus-specific function may be required to obtain a general picture of insulator function.

In mammals, CTCF is the only insulator protein identified so far, but other factors, such as cohesin have been identified as necessary and essential for the formation of CTCF-mediated long-range interactions [28], [30], [32]. Mammalian CTCF contains eleven zinc-fingers, and it has been shown that different combinations of zinc-fingers could be used to bind different DNA sequences [78]. Thus, in mammals CTCF may play the role of first layer insulator protein, whereas other factors such as cohesin or mediator may play the role of second layer insulator proteins [31].

This model proposing different functional roles for insulator factors could also explain the mechanism by which insulators are able to help establish and reinforce the transcriptional state of chromatin domains throughout cell division. First layer proteins remain bound to chromatin at all stages of the cell cycle [15], [79]. In contrast, both CP190 and Chromator are chromatin-bound during interphase but display a drastic redistribution during mitosis: CP190 strongly binds to centrosomes while Chromator co-localizes to the spindle matrix [22], [43]. Thus, the dissociation and cellular redistribution of second layer insulator proteins during cell division would be responsible for the massive remodeling of chromosome architecture occurring during mitosis, and for the re-establishment of higher-order contacts at the onset of interphase. In contrast, first layer insulator proteins would act as anchor points for the re-establishment of higher-order interactions after mitosis, and for the maintenance of the transcriptional identity of physical domains. Thus, our model suggest distinct roles for insulator binding proteins and co-factors in actively shaping the organization of chromatin into physical domains during the cell cycle. This model is consistent with recent genome-wide data suggesting that, overall, first layer insulator proteins remain bound to their binding sites during mitosis, whereas second layer insulator proteins tend to show a large change in binding patterns [79], [80]. Further genome-wide and microscopy experiments will be needed to quantitatively test this model.

## Materials and Methods

### Bacterial strains and protein expression and purification

DNA plasmids were propagated in *E. coli* DH5a or in DB3.1 cells (depending on vector used). Proteins were expressed and purified from E. coli BL21 (DE3)-pLysS cells (Invitrogen) as described elsewhere [81]. Details on vectors, primers, protein constructs and protein purification procedures can be found in Text S1 and in Supplementary Tables S2, S3.

### Electric mobility shift assay (EMSA) and super-shift analysis

A 447 bp genomic region containing the Tudor-SN locus was subcloned into pTST101 to make pTST101-447pos (oligonucleotides are shown in Supplementary Table S4). pTST101-447pos was digested by NdeI, HindIII, and SalI resulting in three linear fragments, including DNA_tudor_ (1627 bp long dsDNA fragment containing the 447 bp Tudor-SN locus) and two additional dsDNA fragments (750 and 4025 bp). Restricted pTST101 (1.7 nM) was incubated with increasing amounts of purified BEAF32, CP190 or Chromator in 150 mM NaCl, 30 mM Tris/HCl pH 7.4, 5 mM mercaptoethanol. A gel loading buffer (50% glycerol, 50 mM Tris/HCl pH 7.4) was added and the DNA-protein mixture was directly analyzed in a 1% TAE agarose gel. DNA was labeled using Sybersafe (Invitrogen) and visualized on a gel imaging system (Image Station 4000 MM Pro–Carestream Molecular Imaging). No difference in binding specificity was observed when DNA competitors (e.g. dIdC) were added to the protein-DNA mix. For super-shift assays, the 447 bp *Tudor-SN* locus (chromosome 3L: 264375–264822) was PCR amplified from S2 Drosophila genomic DNA. Purified proteins were added to the DNA in a reaction mixture in a total volume of 20 µl and incubated for 10 min on ice. A gel loading solution (50% glycerol, 50 mM Tris/HCl pH 7.4) was added and the DNA-protein mixture was directly analyzed on a 2% TAE agarose gel.

### Fluorescence anisotropy

Fluorescence anisotropy experiments used short, 5′-Cy3B labeled DNA fragments (DNA_S_ and DNA_NS_, Eurogentec, oligonucleotide sequences are shown in Supplementary Table S5). Anisotropy measurements were carried out using a Tecan Safire II micro plate reader fluorimeter and a Corning 384 Low Flange Black Flat Bottom plate. All measurements were carried out in 30 mM Tris/HCl pH 7.5, 0.01 mg/ml BSA, 0,004% Tween20, 100 mM NaCl, 20 µM ZnSO_4_, 5 mM mercaptoethanol in a final volume of 60 µl. DNA binding studies were performed by adding increasing amounts (0–800 nM) of purified proteins to 2.5 nM of Cy3B or atto-655 5′-labeled 58-bp dsDNA. Dissociation measurements were performed by adding large amounts (up to 1000 nM) of unlabeled DNA_S_ or NaCl (350 mM final). Further details can be found in Text S1.

### Fluorescence correlation spectroscopy

Reaction buffers and DNA substrates (at a final DNA concentration of 2.5 nM) were the same as those used for fluorescence anisotropy (oligonucleotide sequences are shown in Supplementary Table S5). Fluorescence correlation and cross-correlation experiments were carried out on a custom-built setup allowing Pulse Interleaved Excitation (PIE) with Time Correlated Single Photon Counting (TCSPC) detection as described elsewhere [53]. It is important to note that our measurements allow us to detect only 50% of the complexes involved in bridging, as complexes containing two DNA molecules with the same color do not contribute to the cross-correlation amplitude. Further details on PIE-FCS and the models used to fit data can be found in Text S1.

### Nuclear extracts

Drosophila S2 cells (DGRC) were grown in Schneider cell medium supplemented with 10% calf serum. 3×10^6^ cells were centrifuged for 10 min at 1000 g and 4°C. All subsequent steps were performed on ice. Cells were washed twice in PBS and resuspended in hypotonic lysis buffer (10 mM Tris/HCl pH 7.5,10 mM KCl, 1.5 mM MgCl_2_, complete EDTA-free protease inhibitors (Roche)), and washed again twice with hypotonic buffer. After 30 min on ice, lysed cells were pushed through a 25G needle. In addition, lysates were washed with hypotonic buffer and centrifuged at 1000 g. Nuclei were resuspended in nuclear lysis buffer (300 mM KCl, 50 mM Tris/HCl Ph 7.5,10% glycerol, 1% Triton ×100, and protease inhibitors) with benzonase (Novagen, 71206) and incubated for 30 min on a rotating wheel at 4°C. Next, nuclear lysates were centrifuged at 14000 g for 15 min at 4°C. The supernatant was transferred to a clean tube. This resulted in 200 µl of nuclear extract with a total protein concentration of ∼20 mg/ml. This protocol was adapted from Hart *et al.* [82].

### Western blot analysis

Purified proteins/S2 nuclear extracts were separated on a 10–12% SDS-Polyacrylamide-gel and electro-blotted for 1 h at 100 mV onto a nitrocellulose membrane (Protran* Nitrocellulose Membrane Filters, Whatman*). Next, membranes were blocked (3% BSA in TBST) for 1 h and subsequently washed (1% BSA in TBST) before incubation for 1 h with polyclonal purified primary antibody (guinea-pig-anti-Chromator/rabbit-anti-CP190 or mouse-anti-BEAF32 from DSHB). Several washing steps (1% BSA in TBST) followed before the incubation with HRP-labeled secondary antibody (goat anti-guinea pig IgG-HRP Conjugate Thermo scientific, Goat anti-Mouse IgG (H+L)-HRP conjugate Pierce, goat anti-rabbit IgG (H+L)-HRP Conjugate Biorad) for 40 min. After further washing steps the membrane was developed using Pierce ECL Western Blotting Substrate and imaged (Image Station 4000 MM Pro – Carestream Molecular Imaging).

### CO-IP

Purified polyclonal antibodies (anti-Chromator (60 µg), anti-CP190 (60 µg), control goat-IgG (90 µg) were immobilized (2 h, room temperature) on 100 µl Amino Link Plus Coupling agarose-bead-slurry (Pierce Co-Immunoprecipitation Co-IP Kit) following the manufacturer instructions. Different concentrations of heterologous purified proteins or 100 µl of S2 nuclear extract (20 mg/ml) including protease inhibitor (Roche, EDTA free) were added for control goat-IgG, guinea-pig-anti-Chromator, or rabbit-anti-CP190 immobilized agarose beads in IP-Lysis buffer (part of the Cp-IP Pierce kit, total volume 400 µl) and incubated on a rotary wheel for 1–3 h at 4°C in a final volume of 400 µl. Depending on the bait protein used, the bead-antibody-protein-complex was washed several times with 400 µl IP lysis-buffer, followed by PBS including 200–1000 mM NaCl until no protein could be detected in the washing step. Elution was carried out after incubating the protein-bead complex for 3 min in elution buffer at pH 2.8. Eluted proteins were analyzed by Western-blot-analysis.

### Genome-wide data analysis

Aggregation plots were obtained from genome-wide data from Sexton *et al.* [13], and were constructed by following the strategy developed by Jee *et al.* [83]. First, interacting Hi-C DpnII bins containing genomic features of interest (BEAF32, CP190 or Chromator) were identified. BEAF32 binding sites were considered as anchors and CP190, Chromator or both sites as targets [83]. Second, only LRI at distances between 15 and 60 kbp and containing BEAF32 in the anchor and CP190/Chromator in the target were further considered. The lower limit was set to 15 kbp, as significantly high background levels occur for bins at distances <15 kbp. The upper limit (60 kbp) was set to be smaller than the average size of topological domains [13]. Third, Hi-C interaction profiles were binned in 500 bp windows +/−5 kbp around the target site. Next, target sites were aligned, aggregated together, and normalized (blue solid lines, Figure 6E). Internal controls (grey lines, Figure 6E) were obtained by using the same procedure but for target sites that did not contain any of the features (CP190 or Chromator). This procedure generated background interaction levels reflecting the chromatin context of the anchor site. Frequencies of interactions were statistically tested by Wilcoxon tests.

For the analysis of ChIP-chip data (Venn diagrams), publicly available .gff3 files were downloaded from the modENCODE website (http://data.modencode.org/) corresponding to CP190, BEAF32 and Chromator/Chriz ChIP-chip experiments performed in BG3 and S2 cells [48], [84] (datasets 274, 275, 278, 279, 280, 921, 924). Overlaps between binding sites were calculated with the intersectBed function of the BEDTools software [85]. Venn diagrams were generated with the vennDiagram package in R.

### Crystallization, data collection, processing, structure determination and refinement

Crystallization trials was carried out by the sitting-drop technique using the classic, PEG, PACT and AmSO4 suites (Quiagen, France) and low-profile microplates (Grenier, France) at room temperature. 0.5 µl protein solution was mixed with an equal volume of reservoir solution. Several conditions yielded crystals. Optimizations were done with the hanging-drop vapor diffusion technique. 1 µl protein solution was mixed with 1 µl of reservoir. We obtained well diffracting crystals (2.03 Å) using 0.8 M NaH_2_PO_4_, 0.8 M KH_2_PO_4_, 0.1M Hepes/pH 7.5. Crystals were soaked in 30% glycerol for cryoprotection and diffraction data were collected under cryogenic conditions on our laboratory anode and at the European Synchrotron Radiation Facility (ESRF, Grenoble). Image data were processed and scaled using the programs MOSFLM (Leslie, 1999) and SCALA of the CCP4 suite [86]. The crystal belonged to space group P3_2_21 with unit cell parameters a = b = 84.98 Å, c = 40.87 Å, α = β = 90° and γ = 120°.

The structure of CP190-BTB/POZ was solved by molecular replacement with an in-house dataset at 2.3 Å resolution using the program PHENIX (phenix.autoMR) [87] and a combination of five partial models extracted from the server TOME [88] used to gather potential templates through fold-recognition. Structure refinement and rebuilding were performed with COOT [89], PHENIX (phenix.refine) [87] and REFMAC (Murshudov et al, 1997) from the CCP4 suite [86] using a dataset recorded at the ESRF at 2.0 Å resolution. Data collection and refinement statistics are summarized in Supplementary Table S1. The structure has been deposited with the Protein Data Bank (PDB 4U77).

## Supporting Information

Figure S1 We investigated the stability of protein-DNA complexes by competitive inhibition measurements. BEAF32/CP190/Chromator-DNA_S_ complexes were pre-formed by incubating DNA_S_ (2.5 nM) with saturating amounts of BEAF32, CP190, or Chromator for 5 min at 4°C. Pre-formed complexes were titrated with increasing concentrations of unlabeled DNA_S_, and complex dissociation was monitored using the fluorescence anisotropy signal from Cy3B-DNA_S_. BEAF32, CP190, and Chromator were efficiently competed by DNA_S_ (Supplementary Figure S1A–C). A three-parameter hyperbolic decay curve was used to extract the half-maximal effective concentration (EC_50_), which was used to estimate the apparent equilibrium constant of the competitor (*K_i_*) (Equations S1 and S2, Text S1). Apparent constants were 20±4 nM for BEAF32, 17±4 nM for CP190, and 514±360 nM for Chromator, consistent with our direct equilibrium dissociation constant measurements and indicating that while BEAF32 and CP190 bind DNA with a good affinity, Chromator displays a very poor affinity for DNA. Differences in apparent constants are likely due to this method producing considerable overestimations of the apparent equilibrium constants [1]. Non-fluorescent competitor DNA_S_ was added to a pre-formed complex made by 2.5 nM of Cy3b-labeled DNA_S_ incubated with: (**A**) 100 nM of BEAF32, (**B**) 200 nM CP190, or (**C**) or 638 nM Chromator. Solid lines represent hyperbolic decay fits (see Text S1). (**D**) BEAF32 binding stability on DNA_S_ is monitored while adding increasing concentrations of CP190-BTB/POZ, BSA or MBP. Monovalent salt (350 mM NaCl) was added at the end of the measurement to verify that BEAF32 was still bound to DNA_S_. No relevant DNA binding capability could be observed for CP190-BTB/POZ, BSA or MBP at those concentrations (open symbols). Solid and dashed lines are guides to the eye.
(TIFF)

Figure S2 (A) Anti-CP190 recognize neither BEAF32 nor Chromator. Western blot using anti-CP190 antibody of (1) BEAF32, (2) CP190 (fraction 1), (3) CP190 (fraction 2), (4) Chromator (fraction 1) and (5) Chromator (fraction 2). Anti-CP190 is only specific to CP190. (B) Western blot (using anti-BEAF32 antibody) shows that purified BEAF32 does not bind to anti-CP190, anti-Chromator, or anti-IgG columns. Co-IPs were performed with purified BEAF32 (well 1) run on different co-IP with immobilized: anti-Chromator (well 2), anti- CP190 (well 3) or anti-IgG antibodies (well 4). BEAF32 was not retained by any of the columns. (C–I) Full bands from Co-IPs shown in Fig. 3A–G. See caption of Figure 3 for full details. (J) Interactions between CP190-BTB/POZ and Chromator. CP190-BTB/POZ was Cy5-labelled on its N-terminal. Fluorescence anisotropy of CP190-BTB/POZ-Cy5 was used as a reporter of Chromator binding. The binding of Chromator to CP190-BTB/POZ (blue circles) seems to occur with an apparent affinity of ∼50 nM. Solid blue line is a guide to the eye. The overall small change in anisotropy is due to the relatively small changes in rotational diffusion of CP190-BTB/POZ upon Chromator binding. (K) Co-IP assay with heterologously purified CP190 and Chromator. Goat-IgG or purified rabbit polyclonal antibodies against CP190 were covalently coupled to agarose beads. CP190 and Chromator were incubated and analyzed by SDS-PAGE followed by Western-Blot- analysis (with anti-CP190 antibody for lanes 1–3 and anti-Chromator antibody for lane 4). Lane 1 shows the un-purified mix between CP190 and Chromator. Lane 3 shows that CP190 is not bound by the anti-goat-IgG antibody. Both CP190 (lane 2) and Chromator (lane 4) remain bound to a rabbit anti-CP190 column, suggesting a direct interaction between these proteins. Note that Chromator is not recognized by anti-CP190 (Supplementary Fig. S2B).
(TIFF)

Figure S3 Fluorescence fluctuation analysis of BEAF32, CP190 and Chromator binding to DNA_S_-atto655. Normalized auto-correlations for BEAF32, CP190, CP190-C, Chromator, Chromator-C and their combination using a 2.5 nM atto655-DNA_S_ dsDNA fragment instead of the cy3B-DNA_S_ probe used in Figure 5. Data show similar protein binding (A,C) and interaction (B,D) behaviors as those shown in Figure 5. Protein concentrations used: (A–B) 400 nM BEAF32, 50 nM CP190, 50 nM CP190-C. (C–D) 800 nM BEAF32, 100 nM Chromator, 100 nM Chromator-C.
(TIFF)

Figure S4 Titration of B32S with CP190-C, Chromator-C, or full-length CP190 does not lead to BEAF32 dissociation from DNA. (A) BEAF32 binding stability on DNA_S_ was monitored by following the fluorescence anisotropy signal of a B32S complex while adding increasing concentrations of CP190-C or Chromator-C. Salt (350 mM final NaCl concentration) was added at the en d of the titration as a positive control to verify that the anisotropy signal was specifically reporting on DNA_S_-bound BEAF32 complexes. No DNA binding could be detected for neither CP190-C nor Chromator-C at the same concentrations. (**B**) EMSA using the same DNA fragments than in Figure 2 show that preferential binding of BEAF32 to the specific fragment (lanes 2 and 3, red arrow) is not perturbed by the presence of CP190 (lane 5). Protein concentrations used: 100 and 200, and 200 nM BEAF32 (lanes 2, 3 and 5, respectively), 50 nM CP190 (lanes 4 and 5).
(TIFF)

Figure S5 Venn diagrams showing the genome-wide overlap between (A) BEAF-32, CP190 and Chromator in BG3 cells, and (B) BEAF-32, dCTCF and Chromator in S2 cells calculated from publicly available modENCODE ChIP-chip data [2], [3]. There is a considerably smaller number of BEAF32 peaks in BG3 cells than those observed in other cell types, however the trend of association with CP190 and Chromator remains the same in both S2 and BG3 cell types.
(TIFF)

Figure S6 CP190 binding isotherms for DNA_NS_ (open diamonds) and a DNA fragment of the same length but with the consensous sequence of CP190 [25] (T**GACAC**TG, open squares). Solid lines represent guides to the eye.
(PDF)

Figure S7 Normalized auto-correlation of DNA_S_-Cy3B (black), B32S (red), DNA_S_-Cy3B and CP190-BTB/POZ (light green), and a mix of B32S with CP190-BTB/POZ (dark green). The diffusion time of B32S is unchanged by the addition of CP190-BTB/POZ, suggesting that these domains do not interact directly.
(PDF)

Table S1 Data collection and refinement statistics of the CP190-BTB/POZ crystal structure.
(PDF)

Table S2 Constructs for protein expression and EMSA. BEAF32, CP190, CP190-C, Chromator, Chromator-C were amplified from Drosophila genomic S2 cDNA.
(PDF)

Table S3 Oligonucleotides used for the construction of expression vectors. (Sequences are given 5′- 3′).
(PDF)

Table S4 Oligonucleotides for the construction for pTST-447pos.
(PDF)

Table S5 5′labelled oligonucleotides used for anisotropy and FCCS measurements.
(PDF)

Table S6 Tracks used for Figure 1C.
(PDF)

Text S1 Supplementary Methods and Materials used are described in detail, including protein constructs, expression and purificationation; fluorescence anisotropy competition methods; and fluorescence correlation spectroscopy materials and methods.
(PDF)
