# Supplementary figures and images for: Chromatin Insulator Factors Involved in Long-Range DNA Interactions and Their Role in the Folding of the Drosophila Genome
Source: PLoS Genet. 2014 Aug 28;10(8):e1004544. doi: 10.1371/journal.pgen.1004544 (PMC4148193; doi:10.1371/journal.pgen.1004544)

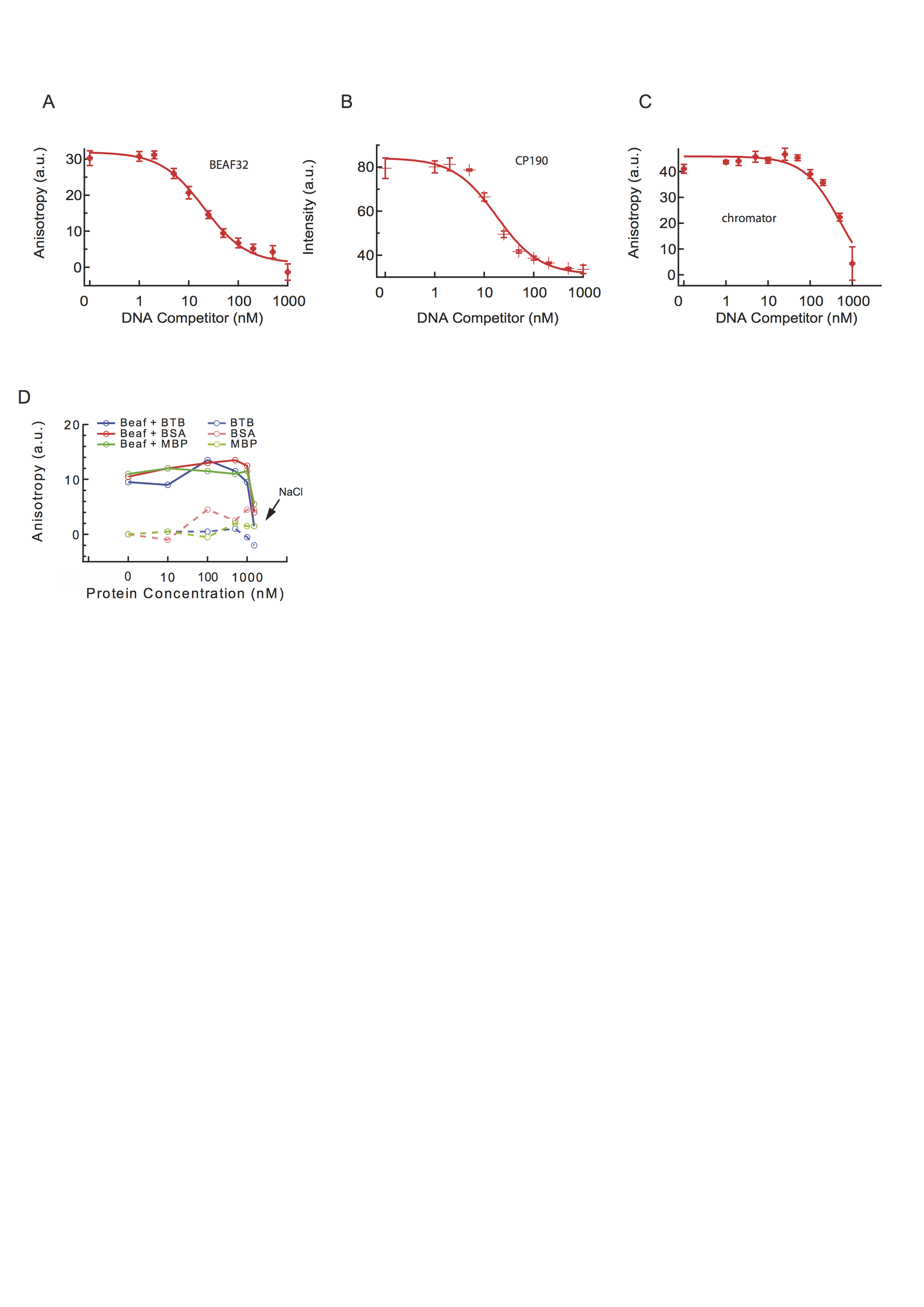

Supplement: Figure S1 — We investigated the stability of protein-DNA complexes by competitive inhibition measurements. BEAF32/CP190/Chromator-DNAS complexes were pre-formed by incubating DNAS (2.5 nM) with saturating amounts of BEAF32, CP190, or Chromator for 5 min at 4°C. Pre-formed complexes were titrated with increasing concentrations of unlabeled DNAS, and complex dissociation was monitored using the fluorescence anisotropy signal from Cy3B-DNAS. BEAF32, CP190, and Chromator were efficiently competed by DNAS (Supplementary Figure S1A–C). A three-parameter hyperbolic decay curve was used to extract the half-maximal effective concentration (EC50), which was used to estimate the apparent equilibrium constant of the competitor (Ki) (Equations S1 and S2, Text S1). Apparent constants were 20±4 nM for BEAF32, 17±4 nM for CP190, and 514±360 nM for Chromator, consistent with our direct equilibrium dissociation constant measurements and indicating that while BEAF32 and CP190 bind DNA with a good affinity, Chromator displays a very poor affinity for DNA. Differences in apparent constants are likely due to this method producing considerable overestimations of the apparent equilibrium constants [1]. Non-fluorescent competitor DNAS was added to a pre-formed complex made by 2.5 nM of Cy3b-labeled DNAS incubated with: (A) 100 nM of BEAF32, (B) 200 nM CP190, or (C) or 638 nM Chromator. Solid lines represent hyperbolic decay fits (see Text S1). (D) BEAF32 binding stability on DNAS is monitored while adding increasing concentrations of CP190-BTB/POZ, BSA or MBP. Monovalent salt (350 mM NaCl) was added at the end of the measurement to verify that BEAF32 was still bound to DNAS. No relevant DNA binding capability could be observed for CP190-BTB/POZ, BSA or MBP at those concentrations (open symbols). Solid and dashed lines are guides to the eye. (TIFF) [file pgen.1004544.s001.tiff]

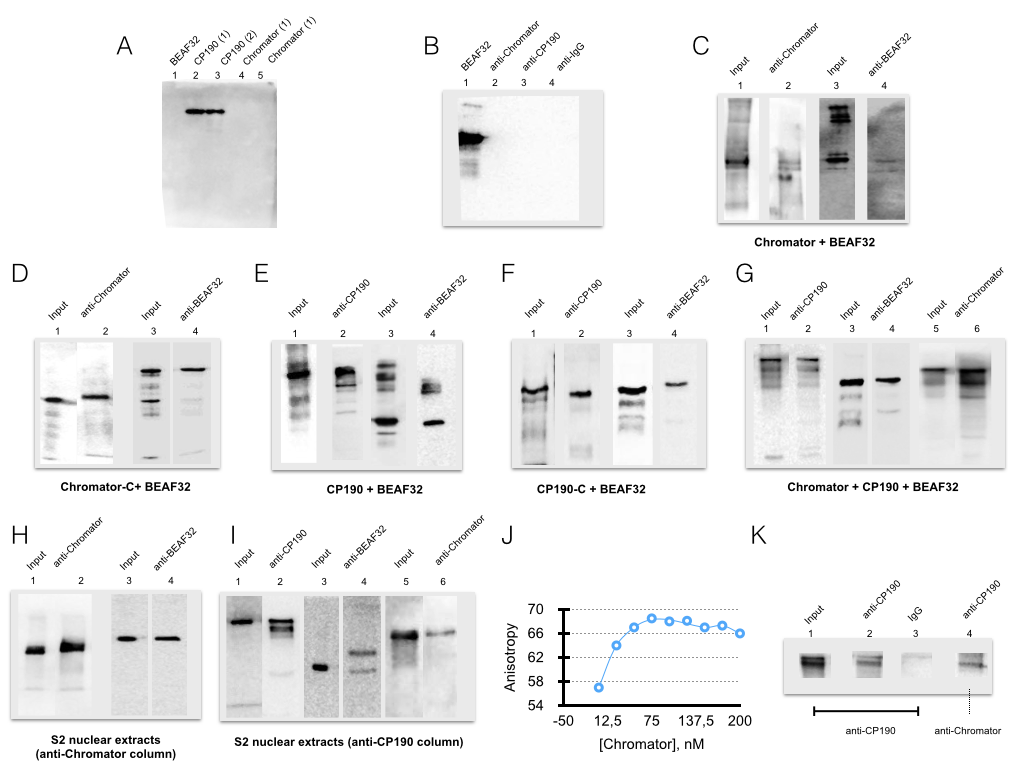

Supplement: Figure S2 — (A) Anti-CP190 recognize neither BEAF32 nor Chromator. Western blot using anti-CP190 antibody of (1) BEAF32, (2) CP190 (fraction 1), (3) CP190 (fraction 2), (4) Chromator (fraction 1) and (5) Chromator (fraction 2). Anti-CP190 is only specific to CP190. (B) Western blot (using anti-BEAF32 antibody) shows that purified BEAF32 does not bind to anti-CP190, anti-Chromator, or anti-IgG columns. Co-IPs were performed with purified BEAF32 (well 1) run on different co-IP with immobilized: anti-Chromator (well 2), anti- CP190 (well 3) or anti-IgG antibodies (well 4). BEAF32 was not retained by any of the columns. (C–I) Full bands from Co-IPs shown in Fig. 3A–G. See caption of Figure 3 for full details. (J) Interactions between CP190-BTB/POZ and Chromator. CP190-BTB/POZ was Cy5-labelled on its N-terminal. Fluorescence anisotropy of CP190-BTB/POZ-Cy5 was used as a reporter of Chromator binding. The binding of Chromator to CP190-BTB/POZ (blue circles) seems to occur with an apparent affinity of ∼50 nM. Solid blue line is a guide to the eye. The overall small change in anisotropy is due to the relatively small changes in rotational diffusion of CP190-BTB/POZ upon Chromator binding. (K) Co-IP assay with heterologously purified CP190 and Chromator. Goat-IgG or purified rabbit polyclonal antibodies against CP190 were covalently coupled to agarose beads. CP190 and Chromator were incubated and analyzed by SDS-PAGE followed by Western-Blot- analysis (with anti-CP190 antibody for lanes 1–3 and anti-Chromator antibody for lane 4). Lane 1 shows the un-purified mix between CP190 and Chromator. Lane 3 shows that CP190 is not bound by the anti-goat-IgG antibody. Both CP190 (lane 2) and Chromator (lane 4) remain bound to a rabbit anti-CP190 column, suggesting a direct interaction between these proteins. Note that Chromator is not recognized by anti-CP190 (Supplementary Fig. S2B). (TIFF) [file pgen.1004544.s002.tiff]

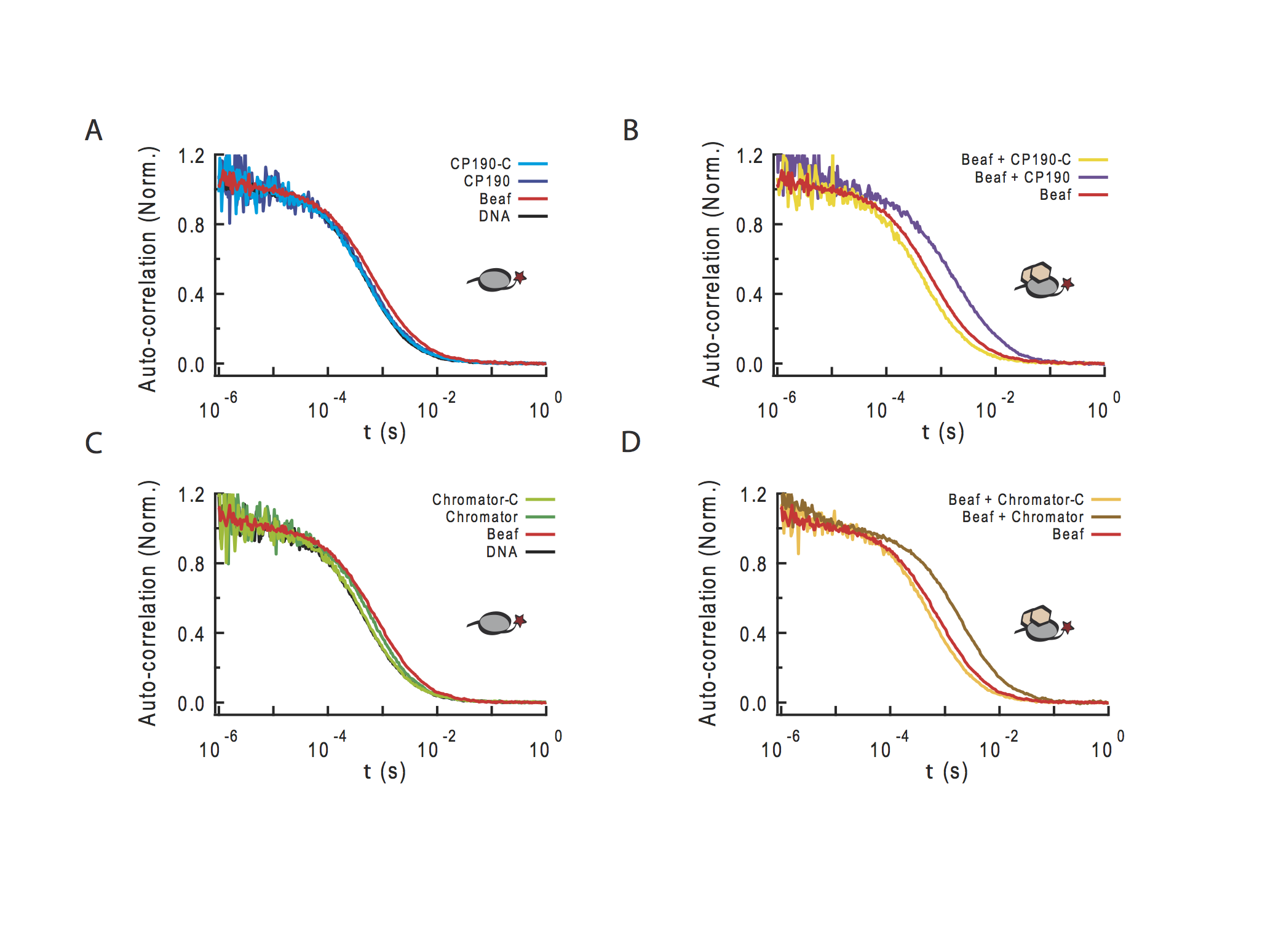

Supplement: Figure S3 — Fluorescence fluctuation analysis of BEAF32, CP190 and Chromator binding to DNAS-atto655. Normalized auto-correlations for BEAF32, CP190, CP190-C, Chromator, Chromator-C and their combination using a 2.5 nM atto655-DNAS dsDNA fragment instead of the cy3B-DNAS probe used in Figure 5. Data show similar protein binding (A,C) and interaction (B,D) behaviors as those shown in Figure 5. Protein concentrations used: (A–B) 400 nM BEAF32, 50 nM CP190, 50 nM CP190-C. (C–D) 800 nM BEAF32, 100 nM Chromator, 100 nM Chromator-C. (TIFF) [file pgen.1004544.s003.tiff]

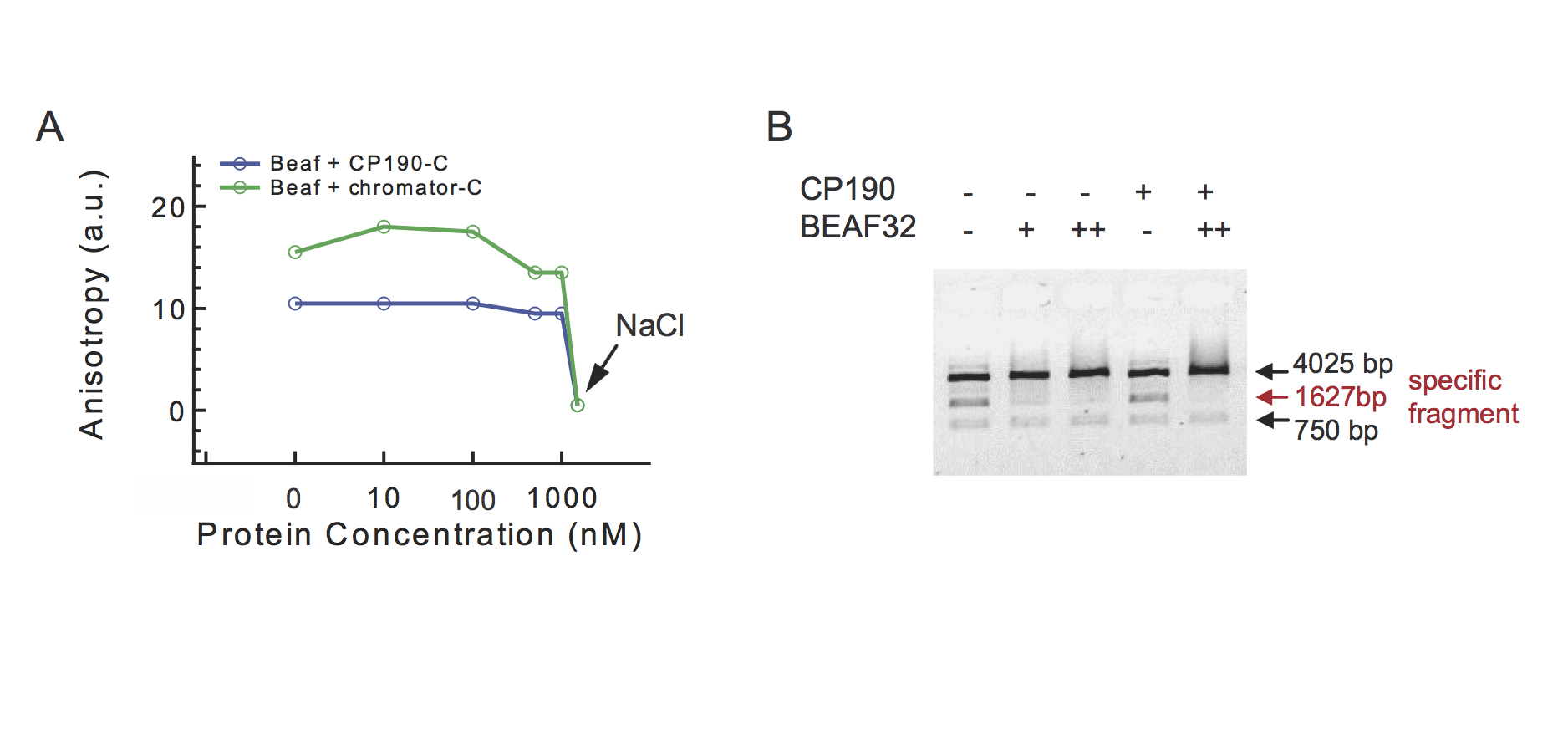

Supplement: Figure S4 — Titration of B32S with CP190-C, Chromator-C, or full-length CP190 does not lead to BEAF32 dissociation from DNA. (A) BEAF32 binding stability on DNAS was monitored by following the fluorescence anisotropy signal of a B32S complex while adding increasing concentrations of CP190-C or Chromator-C. Salt (350 mM final NaCl concentration) was added at the en d of the titration as a positive control to verify that the anisotropy signal was specifically reporting on DNAS-bound BEAF32 complexes. No DNA binding could be detected for neither CP190-C nor Chromator-C at the same concentrations. (B) EMSA using the same DNA fragments than in Figure 2 show that preferential binding of BEAF32 to the specific fragment (lanes 2 and 3, red arrow) is not perturbed by the presence of CP190 (lane 5). Protein concentrations used: 100 and 200, and 200 nM BEAF32 (lanes 2, 3 and 5, respectively), 50 nM CP190 (lanes 4 and 5). (TIFF) [file pgen.1004544.s004.tiff]

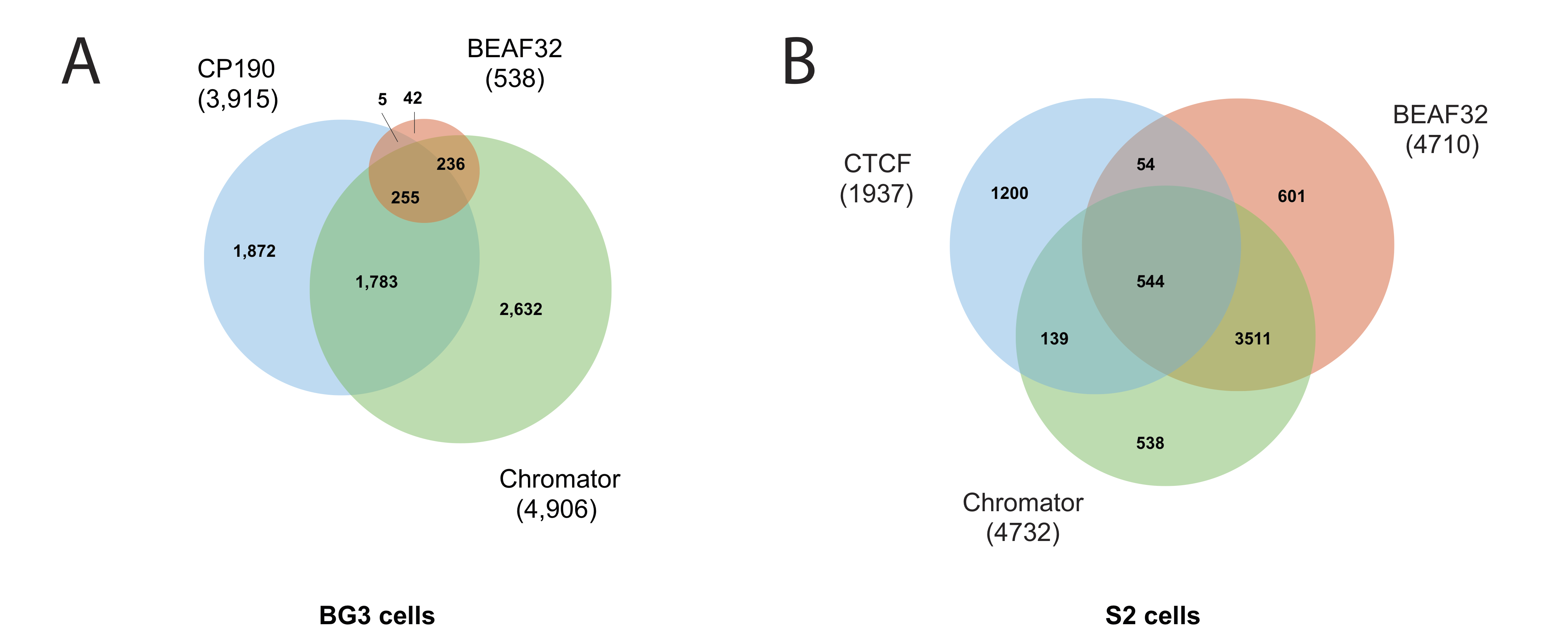

Supplement: Figure S5 — Venn diagrams showing the genome-wide overlap between (A) BEAF-32, CP190 and Chromator in BG3 cells, and (B) BEAF-32, dCTCF and Chromator in S2 cells calculated from publicly available modENCODE ChIP-chip data [2], [3]. There is a considerably smaller number of BEAF32 peaks in BG3 cells than those observed in other cell types, however the trend of association with CP190 and Chromator remains the same in both S2 and BG3 cell types. (TIFF) [file pgen.1004544.s005.tiff]

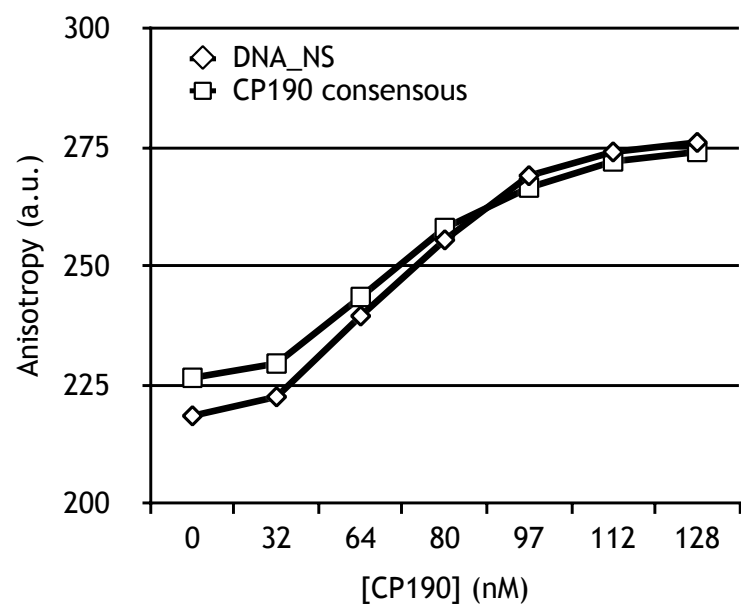

Supplement: Figure S6 — CP190 binding isotherms for DNANS (open diamonds) and a DNA fragment of the same length but with the consensous sequence of CP190 [25] (TGACACTG, open squares). Solid lines represent guides to the eye. (PDF) [file pgen.1004544.s006.pdf]

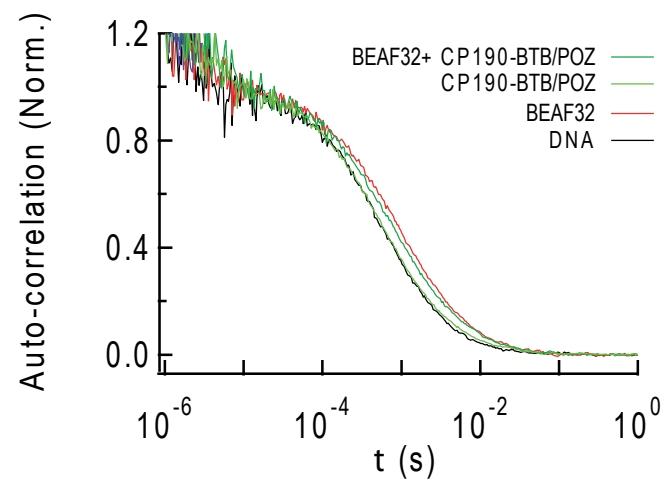

Supplement: Figure S7 — Normalized auto-correlation of DNAS-Cy3B (black), B32S (red), DNAS-Cy3B and CP190-BTB/POZ (light green), and a mix of B32S with CP190-BTB/POZ (dark green). The diffusion time of B32S is unchanged by the addition of CP190-BTB/POZ, suggesting that these domains do not interact directly. (PDF) [file pgen.1004544.s007.pdf]
