# Supplementary material for: Chromatin Insulator Factors Involved in Long-Range DNA Interactions and Their Role in the Folding of the Drosophila Genome
Source: PLoS Genet. 2014 Aug 28;10(8):e1004544. doi: 10.1371/journal.pgen.1004544 (PMC4148193; doi:10.1371/journal.pgen.1004544)
Supplement: Table S1 — Data collection and refinement statistics of the CP190-BTB/POZ crystal structure. (PDF) [file pgen.1004544.s008.pdf]

Crystallographic structure of CP190-BTB/POZ

Supplementary Table 1. Data collection and refinement statistics of the CP190-BTB/POZ crystal structure.

**Data collection**		
Space group	$P3_221$	
Cell dimensions		
a,b,c (Å)	84.98, 84.98, 40.87	
α, β, γ (°)	90, 90, 120	
Resolution, (Å)	42.49 – 2.03 (2.14 – 2.03)*	
R_{merge} %	5.4 (51.6)	
Mean(*I*)/σ*I*	16.6 (3.1)	
Completeness %	100. (100.)	
Redundancy	6.9 (7.1)	
**Refinement**		
Resolution (Å)	2.03	
No of reflections		77479
No unique reflections		11252
R/R_{free}		19.1/24.1
$B_{factors}$ (Å^2)		
protein		60.6
waters		63.8
solvent	63.9	
Rmsd bond lengths (Å)		0.007
Rmsd bond angles °	1.037	
Ramachandran plot (%) (favoured/allowed/disallowed)		98.3/1.7/0.0

* the values in parenthesis are for the highest-resolution shell.
