# Supplementary material for: Chromatin Insulator Factors Involved in Long-Range DNA Interactions and Their Role in the Folding of the Drosophila Genome
Source: PLoS Genet. 2014 Aug 28;10(8):e1004544. doi: 10.1371/journal.pgen.1004544 (PMC4148193; doi:10.1371/journal.pgen.1004544)
Supplement: Table S2 — Constructs for protein expression and EMSA. BEAF32, CP190, CP190-C, Chromator, Chromator-C were amplified from Drosophila genomic S2 cDNA. (PDF) [file pgen.1004544.s009.pdf]

Supplementary Table 2. Constructs for protein expression and EMSA. BEAF32, CP190, CP190-C, Chromator, Chromator-C were amplified from Drosophila genomic S2 cDNA.

**plasmid**	**description**	**reference**
pTST101	E. coli expression vector, bla, malE-egfp	J. Altenbuchner, pers. comm.
pTST101-BEAF-6His	E. coli expression vector, bla, malE-egfp	This study
pTST101-447pos	E. coli expression vector, bla, malE-egfp	This study
pDONR221	Gateway entry vector	Invitrogen
pDONR221- CP190	Gateway entry vector	This study
pDONR221- CP190-C	Gateway entry vector	This study
pDONR221- chromator	Gateway entry vector	This study
pDONR221- chromator-C	Gateway entry vector	This study
pDEST300	Gateway expression vector	Invitrogen
pDEST300-CP190	Gateway expression vector	This study
pDEST300-CP190-C	Gateway expression vector	This study
pDEST300-chromator	Gateway expression vector	This study
pDEST300-chromator-C	Gateway expression vector	This study
