# Supplementary material for: Chromatin Insulator Factors Involved in Long-Range DNA Interactions and Their Role in the Folding of the Drosophila Genome
Source: PLoS Genet. 2014 Aug 28;10(8):e1004544. doi: 10.1371/journal.pgen.1004544 (PMC4148193; doi:10.1371/journal.pgen.1004544)
Supplement: Table S3 — Oligonucleotides used for the construction of expression vectors. (Sequences are given 5′- 3′). (PDF) [file pgen.1004544.s010.pdf]

Supplementary Table 3. Oligonucleotides used for the construction of expression vectors.

(Sequences are given 5´→ 3´)

name	sequence
oh-BglIITEVBEAFFw	AGCGCGAGATCTGAAAACCTGTATTTTCAGGGAATGCC
oh-HindBEAFRev	GGCCGCAAGCTTCAGTGATGATGATGATGATGTCCCTGAAAATACAGGT
TEVBEAFFw	TATTTTCAGGGAATGCCCAAGGGTCGTGTTA
TEVBEAFRev	CTGAAAATACAGGTTTTCCTCATCCTTGGCAAGCGT
ChromatorattB1TEVFw	GGGGACAAGTTTGTACAAAAAAGCAGGCTTCGAAAACCTGTATTTTCAGGGCATGTTGGCACAGGAGATTTCACC
ChromatorattB2Rev	GGGGACCACTTTGTACAAGAAAGCTGGGTGTTACGTTGGGATGTTGAGCGTCTTA
CP190attB1 Fw	GGGGACAAGTTTGTACAAAAAAGCAGGCTTCGAAAACCTGTATTTTCAGGGCATGGGTGAAGTCAAGTCCGTG
CP190attB2Rev	GGGGACCACTTTGTACAAGAAAGCTGGGTGTTATAGCTCCTCCTTCGCCGCCG
chrom-C-601-926attB1	GGGGACAAGTTTGTACAAAAAAGCAGGCTTCGAAAACCTGTATTTTCAGGGCGGAACTCCGCAACATCGCCAAC
chrom-C-attB1	GGGGACAAGTTTGTACAAAAAAGCAGGCTTCATCGATGAAACGGACGAAG
