# Supplementary material for: Chromatin Insulator Factors Involved in Long-Range DNA Interactions and Their Role in the Folding of the Drosophila Genome
Source: PLoS Genet. 2014 Aug 28;10(8):e1004544. doi: 10.1371/journal.pgen.1004544 (PMC4148193; doi:10.1371/journal.pgen.1004544)
Supplement: Table S4 — Oligonucleotides for the construction for pTST-447pos. (PDF) [file pgen.1004544.s011.pdf]

Supplementary Table 4. Oligonucleotides for the construction for pTST-447pos

name	sequence
ohbglIICGATAFw	AGCGCGAGATCTTGACACTGCTAGCTAGAGC
oh-HindIIICGATARev	GGCCGCAAGCTTGGAAATCACGTAACATTCGCCG
