# Supplementary material for: Chromatin Insulator Factors Involved in Long-Range DNA Interactions and Their Role in the Folding of the Drosophila Genome
Source: PLoS Genet. 2014 Aug 28;10(8):e1004544. doi: 10.1371/journal.pgen.1004544 (PMC4148193; doi:10.1371/journal.pgen.1004544)
Supplement: Table S5 — 5′labelled oligonucleotides used for anisotropy and FCCS measurements. (PDF) [file pgen.1004544.s012.pdf]

Supplementary Table 5. 5´labelled oligonucleotides used for anisotropy and FCCS measurements.

**name**	**sequence**
cy3B-DNA$_S$ - Fw	AGAAGTCAGCGCGATAGCATCGATATTTTCGTGACACGCTTGTCATCCGATAGGTAGT
atto655 -DNA$_S$ - Fw	AGAAGTCAGCGCGATAGCATCGATATTTTCGTGACACGCTTGTCATCCGATAGGTAGT
unlabeled DNA$_S$ - Rev	ACTACCTATCGGATGACAAGCGTGTCACGAAAATATCGATGCTATCGCGCTGACTTCT
cy3-DNA$_{NS}$-Fw	GGACAGGTATTGTGCCATACTGACCACATCGTCTTGGTCTATAAGCTCCACGACATCC
unlabeled DNA$_{NS}$-Rev	GGATGTCGTGGAGCTTATAGACCAAGACGATGTGGTCAGTATGGCACAATACCTGTCC
