# Supplementary material for: Chromatin Insulator Factors Involved in Long-Range DNA Interactions and Their Role in the Folding of the Drosophila Genome
Source: PLoS Genet. 2014 Aug 28;10(8):e1004544. doi: 10.1371/journal.pgen.1004544 (PMC4148193; doi:10.1371/journal.pgen.1004544)
Supplement: Table S6 — Tracks used for Figure 1C. (PDF) [file pgen.1004544.s013.pdf]

Supplementary Table 6. Tracks used for Figure 1C

**Protein**	**Track description**
BEAF	BEAF-HB antibody, S2-DRSC, filename: GSM520782_364.Mvalues.bigWig<br>Cell Line S2-DRSC;Antibody BEAF-HB extraction3_array1
Chromator	Chro(Chriz)WR antibody, S2-DRSC, filename:GSM520800_71.Mvalues.bigWig<br>Cell Line S2-DRSC;Antibody Chro(Chriz)WR extraction1_array1
CP190	Cell Line S2-DRSC;Antibody CP190-VC extraction1_array1<br>CP190-VC antibody, S2-DRSC, filename:GSM520804_403.Mvalues.bigWig
CTCF	Cell Line S2-DRSC;Antibody CTCF-VC extraction1_array1<br>CTCF-VC antibody, S2-DRSC, filename:GSM520812_409.Mvalues.bigWig
Su(HW)	Cell Line S2-DRSC;Antibody Su(Hw)-VC extraction3_array1<br>Su(Hw)-VC antibody, S2-DRSC, filename: GSM520985_374.Mvalues.bigWig
