# Supplementary material for: Chromatin Insulator Factors Involved in Long-Range DNA Interactions and Their Role in the Folding of the Drosophila Genome
Source: PLoS Genet. 2014 Aug 28;10(8):e1004544. doi: 10.1371/journal.pgen.1004544 (PMC4148193; doi:10.1371/journal.pgen.1004544)
Supplement: Text S1 — Supplementary Methods and Materials used are described in detail, including protein constructs, expression and purificationation; fluorescence anisotropy competition methods; and fluorescence correlation spectroscopy materials and methods. (PDF) [file pgen.1004544.s014.pdf]

Chromatin insulator factors involved in long-range DNA interactions and their role in the folding of the Drosophila genome

Vogelmann[1*], J., Le Gall[1*], A., Dejardin[1], S., Allemand[1], F., Gamot[2], A., Labesse[1], G., Cuvier[2], O., Nègre[3], N., Cohen-Gonsaud[1], M., Margeat[1], E., Nöllmann [1#], M.

Supplementary Materials and Methods

Protein constructs, expression and purification

Native BEAF32 (BEAF32B, 282 amino acids in length) was cloned into the pTST101 vector, resulting in a construct of 691 amino acids containing a 6xHis C-terminal tag and a MBP-N-terminal tag. Cultures were grown overnight (O/N) from a BEAF32 glycerol stock and grown in Luria Bertani (LB)-medium supplemented with 100µg/ml ampicillin at 37°C. Fresh cultures were started and grown until OD_{595} = 0.7 was reached, then induced with 0.2% rhamnose for 5 h at 25 °C. Cells were harvested by centrifugation at 4°C, and resuspended in lysis buffer (1M NaCl, 20 mM Tris/HCl pH 7.4, protease inhibitor mix (Roche), 10 mM mercaptoethanol). Cells were lysed by French-pressing three times at 1000 psi, and centrifuged at 18000 g for 30 min at 4 °C to separate the soluble protein fraction from cell debris. Supernatant was filtered through a 0.45 µM filter and protein purified via a Dextrin Sepharose High Performance MBPTrap HP (GE Healthcare) column on an Äkta express system. Wash buffer: 150 mM NaCl, 20 mMTris/HCl pH7.4, 5 mM mercaptoethanol. Elution buffer: 150mM NaCl, 20 mMTris /HCl pH 7.4, 20 mM maltose, 5 mM mercaptoethanol).

Native CP190 (1096 amino acids in length) was cloned into the pET300/NT-DEST Gateway vector, resulting in a 1120 amino acids long construct containing a 6xHis N-terminal tag. CP190-C (residues 599 to 1096) was cloned into the pET300 vector, resulting in a 514 amino acids long construct containing a 6xHis N-terminal tag. Native Chromator (926 amino acids in length) was cloned into the pET300 vector, resulting in a 950 amino acids long construct containing a 6xHis N-terminal tag. Chromator-C (residues 601 to 926) was cloned into the pET300 vector, resulting in a 350 amino acids long construct containing a 6xHis N-terminal tag. An *E .coli* BL21(DE3)-pLysS O/N-pre-culture was started from a CP190, CP190-C, Chromator, or Chromator-C glycerol stock and grown in LB supplemented with 100µg/ml ampicillin at 37°C. Fresh cultures were started and grown until OD_{595}= 0.7-0.9 and induced with 0.2mM IPTG for 3 h at 30 °C (C-terminal fragments) or 5 h at 25°C (full-length proteins). Proteins were purified in an Äkta express or explorer system using a HisTrap HP column (GE Healthcare). Wash buffer: 150 mM NaCl, 30 mM Tris/HCl pH7.4, 20 mM Imidazol, 5 mM mercaptoethanol. Elution buffer: 150 mM NaCl, 30 mM Tris/HCl pH 7.4, 500 mM Imidazol, 5 mM mercaptoethanol. Purification of C-terminal fragments of CP190 and Chromator was followed by a further desalting step (GE Healthcare HiPrep 26/10 Desalting Column). Protein concentrations were estimated by absorbance measurements.

The BTB/POZ domain cDNA sequences, coding amino acids 1 to 134 of Drosophila *CP190* protein, was synthesized (GeneArt-lifetechnologies) and subcloned between the NdeI and XhoI sites of pET22b. The resulting vector pET22b-BTB was transformed in *E.coli* BL21(DE3) cells. For expression of the CP190-BTB/POZ domain, BL21(DE3)/pET22b-BTB was grown at 37°C in LB medium supplemented with 200 µg/ml ampicillin. When turbidity reached A_{600} = 0.6 BTB/POZ expression was induced by addition of 0.5 mM IPTG for three hours. Cells were harvested by centrifugation at 6000 g for 15 min at 4°C. The pellet was then resuspended in 20 mM Tris/HCl pH 7.5, 150 mM NaCl and 2 mM ß-mercaptoethanol (buffer A) and stored at -80°C. Cells were lysed by sonication and insoluble proteins and cell debris were sedimented by centrifugation at 40000 g at 4°C for 30 min. Supernatant was filtered through 0.45 µm filters and loaded onto an affinity column (His Trap FF, GE Lifescience), and equilibrated with buffer A. The column was washed with

20 column volumes of buffer A and protein eluted with a linear 0-100% gradient of buffer B (buffer A supplemented with 0.25 M imidazole). The peak fractions were analyzed by SDS-PAGE. Fractions containing BTB were pooled, concentrated, and loaded onto a size-exclusion column (S75, GE Lifescience) equilibrated with 20 mM Tris pH 7.5, 150 mM NaCl and 2 mM DTT. Fractions containing BTB were pooled and concentrate, by ultrafiltration using a Vivaspin 20 (Sartorius) with a 5 kDa cutoff, to 10 mg/ml.

Fluorescence anisotropy competition experiments

Fluorescence anisotropy experiments used short, 5'-Cy3B labeled DNA fragments (DNA_S and DNA_{NS}, Eurogentec), with the following sequences (58 bp, CGATA motifs are shown in bold):

DNA_S sequence: 5'-AGA AGTCAGCG**CGATA**GCAT**CGATA**TTTTCGTGACACGC TTGTCATC**CGATA**GGTAGT.

DNA_{NS} sequence: 5'-GGACAGGTATTGTGCCATACTGACCACATCGTCTTGGTCTAT AAGCTCCACGACATCC. Oligonucleotides were annealed by preparing a 2 µM stock solution in 200 mM Tris/HCl pH 8.4, 500 mM KCl. The mix was incubated for 5 min at 95 °C, wrapped in aluminum foil, and allowed to reach room temperature.

Equilibrium binding isotherms were empirically fitted using either a single site binding model [@Cattoni:2013hx] described by:

$$r_A = \frac{-r_{\max}}{2[B]}\left\{([A]+[B]+K_D)-\left[([A]+[B]+K_D)^2+4[B][A]\right]^{\frac{1}{2}}\right\}$$ Eq. 1

or a multiple site binding model using the Hill equation [@Goutelle:2008vt]:

$$r_A = \frac{r_{max}[A]^n}{K_D^n+[A]^n}$$ Eq. 2

where r_A is the measured anisotropy of the oligonucleotide probe (B), [A] and [B] are the concentrations of protein and probe, respectively, r_{max} represents the final anisotropy value when the oligonucleotide probe is fully bound, K_D represents the apparent dissociation constant and n the Hill coefficient. Data sets were fitted using Igor Pro.

Competition assays were conducted by adding increasing amounts of unlabeled specific DNA (from 1 to 1000 nM) to the 2.5 nM of Cy3B labeled 58-bp dsDNA pre-associated with the protein of interest. For those measurements the protein concentration was chosen to reach an anisotropy value of 80% of the saturation plateau obtained with the direct affinity constant measurements. Error bars correspond to the standard deviation on three measurements performed in parallel. The obtained curves were fitted to a three-parameter hyperbolic decay [@Cattoni:2013hx]:

$$r([DNA_{comp}]) = r_{min} + \frac{r_{max}.EC_{50}}{EC_{50} + [DNA_{comp}]}$$

(Eq. S1), where *r* is the measured anisotropy of the oligonucleotide probe at concentration of competitor DNA, r_{max} represents the initial anisotropy value in the absence of competitor DNA, r_{min} represents the final anisotropy value when the oligonucleotide probe is dissociated from the protein, represents the half maximal effective concentration.

To obtain an apparent equilibrium binding constant of competitor DNA (Ki) was obtained from EC_{50} by the following equation [@Cattoni:2013hx]:

$$K_i = \frac{EC_{50}}{1 + \frac{[DNA]}{K_D}}$$

(Eq. S2), where [DNA] is the concentration of the cy3B-labeled 58-bp dsDNA pre-associated with the protein of interest and K_D the affinity constant between DNA and protein.

Fluorescence correlation spectroscopy

All experiments were performed at a final DNA concentration of 2.5 nM (for both Cy3B- and atto-655-labeled dsDNA fragments).

Fluorescence correlation and cross-correlation experiments were carried out on a custom-built setup allowing Pulse Interleaved Excitation (PIE) with Time Correlated Single Photon Counting (TCSPC) detection as described elsewhere [@Olofsson:2013ut] In brief, the output of a supercontinuum SC450-4-20MhZ laser source (Fianium, Southampton, UK) is divided by a 50:50 beamsplitter cube in order to generate two excitation beams. Each beam is spectrally filtered using excitation bandpass filters (532/10 for Cy3 and 635/10 for atto655). The first beam is delayed relative to the second by adding an extra path length of 8m using mirrors. The two beams are then recombined using a 50:50 beamsplitter cube and coupled into a single-mode fiber (SMF) to obtain two spatially overlapping excitation sources. Beams are collimated using a 10x microscope objective lens and reflected into a Plan Apochromat 100x, NA1.4 objective (Carl Zeiss, Germany) by a dichroic mirror (FF545/650-Di01, Semrock, Rochester, NY, USA).

Emitted photons are collected by the same objective and focused into a 75 µm pinhole. Fluorescence emission is re-collimated and split using a beamsplitter cube. In each channel, photons are spectrally separated using a dichroic mirror (BS-649, Semrock, Rochester, NY, USA) and filtered using emission bandpass filters (ET BP 585/65 [channels 1a and 1b] and ET BP-700/75 [channels 2a and 2b], Chroma, Bellows Falls, VT, USA). Channel 1 photons are focused onto two MPD-1CTC (MPD, Bolzano, Italy) avalanche photodiodes (APD) whereas channel 2 photons are focused onto two SPCM AQR-14 APDs (Perkin Elmer, Fremont, CA, USA).

Data was collected over 20 s using 50 µW time-averaged excitation power in each channel. Correlation of recorded photon events was performed using a custom-written program in Labview (National Instruments Inc., Austin, TX). By using a lifetime filtering algorithm, unwanted contributions from detector after-pulsing or scattered light to the correlation function was discarded and each photon was correlated to its excitation source to eliminate cross-talk and thus false positive cross-correlation. Before calculation of the correlation functions, eventual aggregates detected in the sample were identified on the time intensity traces as bright spikes and filtered out.

Auto-correlations *G(t)* of the red and green channels were fitted using a 3-D diffusion model with a triplet state:

$$G(t) = \frac{1}{N} \cdot \frac{(1-F) + F.e^{\frac{-t}{Tt}}}{1-F} \cdot \left[\left(1 + \frac{t}{Td}\right)^{-\frac{1}{2}} \cdot \left(1 + \frac{t}{Td.r}\right)^{-\frac{1}{2}} \right] \quad \text{Eq. 3}$$

where *F* is the average fraction of triplet state molecules, *Tt* the triplet lifetime, *Td* the diffusion time and *r* the structure parameter of the detection volume ($r=(z_0/w_0)^2$, z_0 and w_0 being the axial and radial waist of the detection volume). Auto-correlation curves were then normalized by 1/N, the inverse of the average number of labeled molecules in the detection volume.

Cross-correlation of the red and green channels were fitted using :

$$G_c(t) = \frac{1}{N_x} \cdot \left[\left(1 + \frac{t}{Td_x}\right)^{-\frac{1}{2}} \cdot \left(1 + \frac{t}{Td_x.r}\right)^{-\frac{1}{2}} \right] \quad \text{Eq. 4}$$

where N_x is the number of complexes associated with both fluorophores and T_{dx} their diffusion time. Cross-correlation curves were multiplied by the number of molecules found in the red channel, N_{red}, in order to express the degree of binding relative to all red molecules.
